# Microglial AKAP8L: a key mediator in diabetes-associated cognitive impairment via autophagy inhibition and neuroinflammation triggering

**DOI:** 10.1186/s12974-024-03170-z

**Published:** 2024-07-20

**Authors:** Wen-yuan Zhang, Qian-qian Wei, Tao Zhang, Chang-shui Wang, Jing Chen, Jian-hua Wang, Xin Xie, Pei Jiang

**Affiliations:** 1 Department of Pharmacy, Zhongshan City People’s Hospital, Zhongshan, 528403 China; 2 School of Pharmaceutical Sciences, Zunyi Medical University, Zunyi, 510006 China; 3 Translational Pharmaceutical Laboratory, Jining First People ’ s Hospital, Shandong First Medical University, Jining, 272000 China; 4 Institute of Translational Pharmacy, Jining Medical Research Academy, Jining, 272000 China; 5 Department of Neurosurgery, Affiliated Hospital of Jining Medical University, Jining, 272000 China; 6 Neurobiology Key Laboratory, Jining Medical University, Jining, 272067 China; 7 Division of Biomedical Sciences, Warwick Medical School, University of Warwick, Coventry, CV4 7AL UK

**Keywords:** AKAP8L, mTOR, Diabetes-associated cognitive impairment, Microglia, Autophagy, Neuroinflammation

## Abstract

**Background:**

Diabetes-associated cognitive impairment (DACI) poses a significant challenge to the self-management of diabetes, markedly elevating the risk of adverse complications. A burgeoning body of evidence implicates microglia as a central player in the pathogenesis of DACI.

**Methods:**

We utilized proteomics to identify potential biomarkers in high glucose (HG)-treated microglia, followed by gene knockdown techniques for mechanistic validation in vitro and in vivo.

**Results:**

Our proteomic analysis identified a significant upregulation of AKAP8L in HG-treated microglia, with concurrent dysregulation of autophagy and inflammation markers, making AKAP8L a novel biomarker of interest. Notably, the accumulation of AKAP8L was specific to HG-treated microglia, with no observed changes in co-cultured astrocytes or neurons, a pattern that was mirrored in streptozotocin (STZ)-induced diabetic mice. Further studies through co-immunoprecipitation and proximity ligation assay indicated that the elevated AKAP8L in HG-treated microglial cells interacts with the mTORC1. In the STZ mouse model, we demonstrated that both AKAP8L knockdown and rapamycin treatment significantly enhanced cognitive function, as evidenced by improved performance in the Morris water maze, and reduced microglial activation. Moreover, these interventions effectively suppressed mTORC1 signaling, normalized autophagic flux, mitigated neuroinflammation, and decreased pyroptosis.

**Conclusions:**

Our findings highlight the critical role of AKAP8L in the development of DACI. By interacting with mTORC1, AKAP8L appears to obstruct autophagic processes and initiate a cascade of neuroinflammatory responses. The identification of AKAP8L as a key mediator in DACI opens up new avenues for potential therapeutic interventions.

**Supplementary Information:**

The online version contains supplementary material available at 10.1186/s12974-024-03170-z.

## Introduction

Diabetes and its associated complications are significant contributors to the global health burden [1]. Among these, diabetes-associated cognitive impairment (DACI) has become a prominent concern [2]. Clinical research suggests that individuals with diabetes face a 1.73-fold higher risk of developing dementia and a 2.27-fold increased risk of experiencing cognitive impairment [3]. Moreover, DACI poses a challenge to the self-management of diabetes in patients, thereby exacerbating the progression of the disease and increasing the burden of diabetes care. The pathophysiology of DACI is complex and includes factors such as insulin resistance, inflammation, lipid abnormalities, oxidative stress, mitochondrial dysfunction, and autophagy [4]. Despite these insights, the specific mechanisms underlying DACI remain to be fully understood.

A growing body of evidence indicates that microglia play a pivotal role in the development of DACI [5]. In a healthy state, microglia are essential for maintaining brain homeostasis by phagocytically clearing damaged neurons and aggregated misfolded proteins [6]. However, under pathological conditions, such as those present in diabetes, microglia undergo a transition from a protective surveillance state to an overactivated state, which can lead to synaptic damage, neuronal apoptosis, and the suppression of neurogenesis [7]. Exposure of microglia to high glucose (HG) for 24 h triggers a dose-dependent cytotoxicity through increased apoptosis [8]. In animal models of diabetes, there is a notable correlation between cognitive impairment and increased activation of microglia, as indicated by the upregulation of Ionized calcium binding adapter molecule 1 (Iba1), a marker of microglial activation, within the brain [9, 10]. This is complemented by clinical studies that corroborate the findings from basic research [11, 12], thereby reinforcing the notion that microglial activation is a critical factor in the pathogenesis of DACI.

Proteomics has become an indispensable tool in diabetes research, providing valuable insights into the intricate protein networks that can become disrupted due to the disease. This technology has led to the discovery of novel biomarkers such as Growth differentiation factor-15/Macrophage inhibitory cytokine-1 (GDF15/MIC-1), interleukin (IL)-18Ra, and Cysteine rich with EGF like domains 1 (CRELD1), which are indicative of disease progression in diabetes [13]. Furthermore, proteomics has been instrumental in investigating the complications associated with diabetes. For instance, the involvement of astrocyte N-Myc downstream regulated gene 2 (NDRG2) in the amelioration of DACI has been confirmed through proteomic analysis. NDRG2 has been shown to modulate astrocytic-neuronal interactions and restore synaptic function in diabetic mice by regulating the Nuclear factor kappa-B/Complement C3/ Complement C3a Receptor (NF-κB/C3/C3aR) signaling pathway [14]. In the context of vascular dementia in diabetic patients, urine proteomic analysis has validated certain protein markers (including Haptoglobin (HP), Serpin domain-containing protein (SERPIND), ATP Synthase Peripheral Stalk-Membrane Subunit B (ATP5PB), Vanin 2 (VNN2), Complement C6 (C6), among others) using the receiver operating characteristic (ROC) curve method [15].

In this study, we have pioneeringly explored the proteomic landscape within microglia exposed to high glucose conditions. We have successfully identified A-kinase anchor protein 8-like (AKAP8L) as a novel and significant biomarker. Furthermore, by examining the Alzheimer’s Disease (AD) postmortem database, we noted a consistent upregulation of AKAP8L expression in AD patients [16]. Moving forward, our objective is to rigorously validate the functional contributions of AKAP8L and to elucidate the pathways in which it is involved, utilizing both in vivo and in vitro models. Our work is poised to yield innovative biomarker that could pave the way for the development of potential therapeutic strategies targeting DACI.

## Materials and methods

### Cells culture and treatment

Primary microglia were cultured from the brain of 1-day-old mice. Mouse brains were harvested, the meninges were removed, and the brain pieces were dissected into small pieces in Ca^2+^ and Mg^2+^-free HBSS (Hank’s Balanced Salt Solution). The tissue pieces were then incubated at 37 °C for 15 min in 0.125% trypsin solution, with gentle mechanical disruption to facilitate enzymatic digestion. Cells were cultured in Dulbecco’s Modified Eagle’s Medium (DMEM) supplemented with 10% fetal bovine serum, 2 mM L-glutamine, 100 U/mL penicillin, and 0.1 mg/mL streptomycin at 37 °C with 5% CO_2_ for 10–14 days. Following this, the mixed cells in the media were shaken at 200 rpm for 4 h at 37 °C to suspend the loosely attached microglia. The media was centrifuged, and the microglia formed a pellet. In parallel, BV2 murine microglial cells, procured from Procell Life Science & Technology Co., Ltd. (Wuhan, China), were cultured under stringent conditions. These cultures were conducted in a fully humidified atmosphere with 5% CO_2_ at a temperature of 37 °C, utilizing a DMEM/F-12 medium enriched with 10% Fetal Bovine Serum (FBS).

Brain mixed cultures were prepared according to the method outlined by previous literature, with slight adaptations [17]. To begin, brains were harvested from one-day-old mouse pups and enzymatically digested for 1 h at 37 ℃ in a 5% CO_2_ environment using 20 U/mL papain. Following the enzymatic treatment, the papain was removed, and the tissue was gently rinsed in DMEM. The tissue was then mechanically dissociated using finely polished glass serological pipettes until all visible aggregates were dispersed. The dissociated cells were seeded onto multiwell chambers coated with poly-L-lysine and incubated in DMEM supplemented with 10% Fetal Bovine Serum (FBS), 1% glutamine, and 1% penicillin-streptomycin. The cultures were maintained in a humidified atmosphere with 5% CO_2_ at 37 ℃, with the medium being refreshed by replacing half of it with fresh medium every three days.

The experimental design segregated the cells into Control and HG groups. The Control group was maintained under normal glucose conditions at a concentration of 5.5 mM. Conversely, the HG group was challenged with a HG (33 mM; Solarbio, Beijing, China) environment for a duration of 48 h [18]. Further categorization of the BV2 cells yielded four distinct experimental groups: Control, HG, HG + si-AKAP8L, and HG + RAPA. The HG + si-AKAP8L group was pre-treated with AKAP8L small interfering RNA (siRNA) for 24 h, followed by exposure to high glucose (33 mM) for an additional 48 h. The siRNAs specific to AKAP8L were synthesized from GenePharma (Shanghai, China) and transfected into BV2 cells. Cells were seeded in plates one day before transfection to reach about 70% confluency on the day of transfection. The transfection procedure was performed using Lipofectamine 3000 (Invitrogen) according to the manufacturer’s instructions. The HG + RAPA group was initially treated with 100 nM rapamycin for 24 h, after which they were exposed to high glucose (33 mM) for an additional 48 h, following a rapamycin dosage previously reported in the literature [19]. Rapamycin was dissolved in dimethyl sulfoxide (DMSO), and control groups were exposed to an equivalent concentration of DMSO (< 0.2%) to account for any solvent effects.

### Animals and drug treatment

Eight-week-old male C57BL/6 mice were obtained from Jinan Pengyue Laboratory Animal Breeding Co., Ltd. (Jinan, China). The animals were acclimated to standard laboratory conditions (22 ± 1 °C, 55 ± 5% humidity, and a 12-hour light-dark cycle) and had free access to food and water. All procedures were approved by the Animal Ethics Committee of Zhongshan City People’s Hospital, adhering to the ethical standards outlined in the Guide for the Care and Use of Laboratory Animals (Chinese Council).

The animal study consisted of two episodes. In the first episode, the mice were randomly assigned to the Control group and the Streptozotocin (STZ; Yisheng Biotechnology Co., LTD Shanghai, China) group (*n* = 6/group), with the aim of investigating whether AKAP8L is upregulated in hippocampal microglia. In the second episode, to investigate whether inhibition of AKAP8L or mTORC1 with sh-AKAP8L or rapamycin (RAPA; MCE Shanghai, China) could improve DACI, the mice were randomly assigned to the following four groups: the Control group, the STZ group, the STZ + sh-AKAP8L group, and the STZ + RAPA group (*n* = 12/group).

In the STZ-induced diabetic model, C57BL/6J mice were administered an intraperitoneal injection of STZ at a dosage of 50 mg/kg once daily for five consecutive days, with the STZ being dissolved in a 0.01 M sodium citrate buffer at a pH of 4.4. The Control group received an equivalent volume of citrate buffer. Body weight and fasting blood glucose levels were assessed from a tail vein blood sample using an OneTouch^®^ Ultra blood glucose meter. Mice with blood glucose levels above 11.1 mmol/L were designated as the diabetes model group for subsequent experiments.

In preliminary experiments, we observed that AKAP8L knockdown, induced by adeno-associated virus (AAV), had no impact on the body weight and blood glucose levels of mice, as illustrated in Supplementary Fig. 1. In the STZ + sh-AKAP8L group, the selected shRNA sequence and scrambled control were cloned into the pAAV vector under control of U6 promoter (Shandong ViGene Biosciences). To perform the stereotaxic injection, mice were anesthetized with isoflurane and fixed to a stereotaxic apparatus. Subsequently, 1 µL of AAV9-U6-sh-NC (for the Control, STZ, and STZ + RAPA groups) and AAV9-U6-sh-AKAP8L (for the STZ + sh-AKAP8L group) were slowly injected into each side of the hippocampus (Bregma, 2.3 mm; Lateral, 1.8 mm; Vertical, 2.0 mm) of the anesthetized mice. The injection was carried out using a 10-µL Hamilton injector fitted with a 30-gauge beveled needle at a rate of 0.25 µL/minute. Upon completing the injection, the needle was retained in position for 5 min and then gently withdrawn to prevent viral reflux. As referenced in literature [20], rapamycin was administered via intraperitoneal injection at a dosage of 2 mg/kg every other day for a period of two weeks. For preparation, rapamycin was initially dissolved in DMSO and subsequently diluted with a specialized solution comprising 5% DMSO, 30% PEG400, 5% Tween 80, and 60% saline to facilitate in vivo application. Control groups were administered the same vehicle devoid of rapamycin. After anesthesia, half of the mice in each group underwent transcardiac perfusion with cold 0.9% saline containing heparin, followed by 4% paraformaldehyde for brain fixation. The brains were then paraffin-embedded and sectioned at a thickness of 4 μm for staining. The timeline for sh-AKAP8L and rapamycin treatments in STZ mice is depicted in Supplementary Fig. 2.

### Proteomic analysis

Primary microglial cells from both Control and HG groups were subjected to a comprehensive proteomic analysis. Proteins were extracted and underwent a meticulous quality assessment. Subsequently, they were enzymatically digested and labeled with TMT reagent (ThermoFisher), which involves the addition of acetonitrile and hydroxylamine. The labeled proteins were then analyzed using liquid chromatography-tandem mass spectrometry (LC-MS/MS). The resulting mass spectrometry data were cross-referenced with major protein databases, including Uniprot, NR, GO, KEGG, and String, to obtain the subcellular localization and annotation information for each identified protein. To identify differentially expressed proteins between the groups, we established stringent criteria based on Fold Change (FC) and statistical significance. Proteins with FC ≥ 1.5 or FC ≤ 0.66 and *p* < 0.05, were selected as differential proteins. This systematic approach ensures a robust and reliable identification of proteins that may play a significant role in the underlying biological processes.

### Immunofluorescence assay

Immunofluorescence staining was performed on frozen hippocampal sections or cells embedded in paraffin. The samples were washed three times with PBS and then incubated overnight at 4 °C in a humidified atmosphere with primary antibodies. The following primary antibodies were used: anti-IL-1 beta (1:100, Abcam, Shanghai, China), anti-p62 (1:100, Abcam, Shanghai, China), anti-AKAP8L (1:100, Santa Cruz, Shanghai, China), anti-βIII-Tubulin (1:500, Servicebio, Wuhan, China), anti-GFAP (1:500, Servicebio, Wuhan, China), anti-Iba-1 (1:500, Servicebio, Wuhan, China), anti-GSDMD (1:500, Abcam, Shanghai, China), anti-NLRP3 (1:200, Abcam, Shanghai, China), LC3 (1:200, Proteintech, Wuhan, China), NeuN (1:200, Abcam, Shanghai, China) and DAPI (Servicebio, Wuhan, China). After the primary antibody incubation, the sections were incubated with a mixture of secondary antibodies conjugated to Alexa-488 (green, Invitrogen, Shanghai, China), Alexa-594 (red, Invitrogen, Shanghai, China), and Alexa-647 (red, Invitrogen, Shanghai, China) for 2 h at room temperature in the dark. The secondary antibodies included donkey anti-goat, anti-rabbit, and anti-mouse, which were pre-absorbed to minimize cross-reactivity. The sections were then mounted using a medium containing 50% glycerol. Examination was performed on five random fields per section under a fluorescence microscope (Olympus, Tokyo, Japan) by an observer blinded to the experimental groups. For the NeuN staining, three sections per mouse were analyzed. Cells that stained positively for NeuN (NeuN^+^) were considered to represent living neurons, as referenced in the literature [21].

### Quantitative real-time PCR (RT- qPCR)

To assess the mRNA expression levels of IL-1β, tumor necrosis factor (TNF)-α, Cluster of differentiation 86 (CD86), IL-6, Inducible nitric oxide synthase (iNOS), IL-4, Arginase-1 (Arg1), and Transforming growth factor-β (TGF-β), total RNA was extracted from the samples using Trizol reagent (Invitrogen, Shanghai, China). Subsequently, complementary DNA (cDNA) was synthesized utilizing a cDNA library construction kit (Tiangen, Beijing, China). The sequences of the primers used are detailed in Table 1. RT-qPCR was performed on a Bio-Rad CFX96™ detection system using SYBR PCR Master Mix (Tiangen, Beijing, China). The thermal conditions were established as such: a temperature of 95 ℃ for 15 min, and finally, 40 cycles of amplification at a temperature of 95 ℃ for 10 s, and a temperature of 60 ℃ for 30 s. β-actin was used as an internal standard to normalize the relative expression levels. The data for IL-1β, IL-6, CD86, TNF-α, iNOS, IL4, Arg1, and TGF-β are presented as the mean Z-scores from three independent experiments, calculated based on the Ct values and the standard curve method.

**Table 1:** Primer sequences used for the qPCR analysis

Gene	Sense Primer (5’-3’)	Antisense Primer (5’-3’)
TNF-α	GGTGCCTATGTCTCAGCCTC	GCCATAGAACTGATGAGAGG
IL-1β	GTGTCTTTCCCGTGGACCTT	TCATCTCGGAGCCTGTAGTG
IL-6	TACCACTTCACAAGTCGGAG	CTGCAAGTGCATCATCGTTG
CD86	GATGGACCCCAGATGCACCA	GTCTCCACGGAAACAGCA
iNOS	GAGACAGGGAAGTCTGAAGC	CCAGCAGTAGTTGCTCCTCT
IL-4	ATCATCGGCATTTTGAACGAG	ACCTTGGAAGCCCTACAGAC
Arg-1	AGTTGGAAGCATCTCTGGCC	ATCACCTTGCCAATCCCCAG
TGF-β	CCGCAACAACGCCATCTATG	AGCCCTGTATTCCGTCTCCT
β-actin	CATTGCTGACAGGATGCAGA	TGCTGGAAGGTGGACAGTGA

### Co-immunoprecipitation (Co-IP) assay and Western blotting

The Co-immunoprecipitation (Co-IP) assay was performed using a Co-IP kit (Absin Bioscienc, Shanghai, China) according to the manufacturer’s instructions. The BV2 cells were transfected with the FLAG-tagged AKAP8L and HA-tagged Raptor plasmids. All steps were done on ice or at 4 °C. Cells were cold-washed once with 1-fold PBS and then lysed with 0.3% CHAPS. Cell debris was pelleted by centrifugation at 15,000 rpm for 10 min. 75 µL of the lysate was taken for whole cell lysate, and the remaining lysate was used for immunoprecipitation. Proteins were eluted from the beads and separated by sodium dodecyl-sulfate polyacrylamide gel electrophoresis (SDS-PAGE).

For Western blotting, the hippocampal tissues or cultured cells were homogenized, and the total proteins were extracted using RIPA lysis buffer. Protein concentrations were determined using the BCA testing kit. Each sample of 50 µg proteins was separated by electrophoresis on a 12% SDS-PAGE gel. The proteins were transferred to polyvinylidene fluoride (PVDF) membranes and blocked in 10% non-fat milk for 1 h at room temperature. Subsequently, membranes were incubated overnight at 4 °C with the following primary antibodies: anti-LC3 (1:500, Proteintech, Wuhan, China), anti-p62 (1:500, Proteintech, Wuhan, China), anti-Beclin1 (1:500, CST, Shanghai, China), anti-AKAP8L (1:1000, Santa Cruz, Shanghai, China), anti-p-mTOR (Ser2448) (1:1000, Cell Signaling Technology, Shanghai, China), anti-mTOR (1:1000, Cell Signaling Technology, Shanghai, China), anti-p-p70S6K (1:1000, Cell Signaling Technology, Shanghai, China), anti-p70S6K (1:1000, Cell Signaling Technology, Shanghai, China), anti-p-ULK1 (1:1000, Cell Signaling Technology, Shanghai, China), anti-ULK1 (1:1000, Cell Signaling Technology, Shanghai, China), anti-NLRP3 antibody (1:1000, Cell Signaling Technology, Shanghai, China), anti-ASC (1:1000, Cell Signaling Technology, Shanghai, China), anti-Caspase-1 (1:1000, Cell Signaling Technology, Shanghai, China), anti-TXNIP antibody (1:1000, Proteintech, Wuhan, China), anti-N-terminal of GSDMD (1:1000, Abcam, Shanghai, China), anti-GSDMD (1:1000, Abcam, Shanghai, China), anti-IL-1β antibody (1:1000, Abcam, Shanghai, China), and β-actin (81115-1-RR, 1:2000, Proteintech, Wuhan, China). The blots were then washed with Tris-buffered saline-Tween-20 (TBST) for three times, and incubated with secondary antibodies. Finally, the signals were detected with an ECL kit (Thermo Fisher, Shanghai, China). The band intensities were analyzed using Image J software and normalized to the β-actin.

### Measurement of Autophagy Flux

The measurement of autophagy flux is referred to previous literature [22]. The BV2 cells and primary microglia were seeded at a density of 1 × 10^5^ cells/well and cultured overnight, after which cells were transfected with mRFP-GFP-LC3 adenovirus (Hanbio, Shanghai, China) for 24 h according to the manufacturer’s protocol. Cells were fixed with 4% paraformaldehyde and the digital images of LC3-positive puncta were captured by TCS SP8 confocal laser scanning microscope. The number of LC3-positive puncta per cell within each experimental group was quantified using ImageJ software.

### Proximity ligation assay

Proximity Ligation Assay (PLA) was executed utilizing the Duolink PLA kit from Sigma, following the provided protocol [23]. Cells were cytospun onto glass slides, fixed with 4% paraformaldehyde (PFA), and permeabilized with 0.2% Triton X-100 for 5 min. After blocking with Duolink blocking buffer for 30 min, the cells were incubated with primary antibodies against Raptor and AKAP8L overnight at 4℃. PLA probes were mixed with Duolink antibody diluent and applied to the slides, followed by ligation and amplification steps to detect protein complexes. Slides were washed, mounted with Duolink in situ mounting medium containing DAPI to stain nuclei, and visualized under an Olympus fluorescence microscope (Olympus, Tokyo, JPN). This method allowed for the specific detection of protein interactions within the cellular environment.

### Enzyme-linked immunosorbent assay (ELISA)

IL-18 levels in the culture media were determined using an ELISA kit according to the manufacturer’s protocol (R&D Systems, Cat. No. DY7625-05). Plates were read at a wavelength of 450 nm using a TECAN Genios reader (TECAN, Durham, NC, USA).

### Transmission electron microscope

In accordance with a previously published method [24], the cells were fixed with a 2.5% glutaraldehyde solution, washed with PBS three times for 5 min each at room temperature, treated with 1% osmic acid for 1 h, washed again with PBS, and dehydrated through a graded series of propanol. Finally, the cells were embedded in an epoxy resin after being saturated with a 2% uranyl acetate solution. Autophagy and pyroptosis in the different treatment groups were observed in the different treatment groups using transmission electron microscopy (HITACHI, HT7700, Tokyo, Japan).

### Dye uptake

Cells were incubated with YO-PRO-1 iodide at a concentration of 0.2 mM for 15 min. Subsequently, the cells were counterstained with Hoechst 33,342, and images were acquired using an inverted microscope (Olympus, Tokyo, Japan). This technique facilitated the assessment of cellular integrity and viability through the visualization of dye penetration [25].

### Sholl analysis

Sholl analysis was conducted in accordance with the established methodology from a previous study [26]. Confocal images of Iba-1-stained brain sections were obtained using a confocal microscope and imported into FIJI software. The images were thresholded to binary, including the complete processes of microglia. The surrounding processes of other cells were erased using the eraser tool to isolate individual microglia. The longest process was drawn using the line segment tool. The Sholl analysis function was used, defining the first shell at 10 micrometers from the soma center (to exclude the soma from the analysis), with each subsequent shell at 5 micrometers. The software automatically calculates the number of intersections with each concentric shell, considering a linear profile.

### Morris water maze test

The learning and memory capabilities of the mice were assessed using the Morris Water Maze Test (MWM). The MWM consisted of a circular tank, subdivided into four equally sized imaginary quadrants, with a hidden platform submerged 1 cm below the water surface at the center of one quadrant. The navigation trials were conducted over the first 5 days. In each trial, the mouse was placed in the tank facing the wall from one of four different starting points and given 60 s to locate the hidden platform. The time taken to reach the platform was recorded as the escape latency. If the mouse failed to find the platform within the allotted time, it was gently guided to the platform and allowed to remain for 10 s; in such cases, the escape latency was recorded as 60 s. Following a 24-hour period, the escape platform was removed, and the mice were placed in the water maze from the opposite quadrant to conduct the spatial probe test. During this test, the duration the mice spent in the former platform’s quadrant and their swimming velocity were meticulously documented. Visutrack 3.0 (Xinruan, Shanghai, China) was used in behavioral test analysis. These behavioral tests provide a comprehensive assessment of the mice’s cognitive abilities, particularly in relation to learning and memory functions.

### Staining

Following the meticulous guidelines of the HE Staining kit (C1015, Beyotime, Beijing, China), the paraffin-embedded sections were stained with hematoxylin to label nuclei and eosin to label cytoplasm. HE-positive cells in the hippocampus were counted in five random fields per section using a light microscope by an observer who was blinded to the experimental groups, ensuring unbiased quantification. For Nissl staining, the procedure was strictly followed as per the manufacturer’s instructions (Nissl staining kit #G1430, Solarbio, Beijing, China). The total number of Nissl-positive neurons in the penumbra was counted in five different fields of view per section by an observer unaware of the treatment groups, using light microscopy.

### Statistical analysis

The statistical analysis was carried out using SPSS (IBM Corp., Armonk, NY, USA, Version 29.0), and results are presented as means ± standard deviation. Statistical analysis was conducted using unpaired Student’s t-tests and one-way and two-way analyses of variance (ANOVA). In order to compare the groups following ANOVA, a post hoc Tukey test was conducted. A P-value of less than 0.05 was considered statistically significant.

## Results

### Proteomics revealed AKAP8L, a novel marker, in HG-induced microglia

**Fig. 1:**
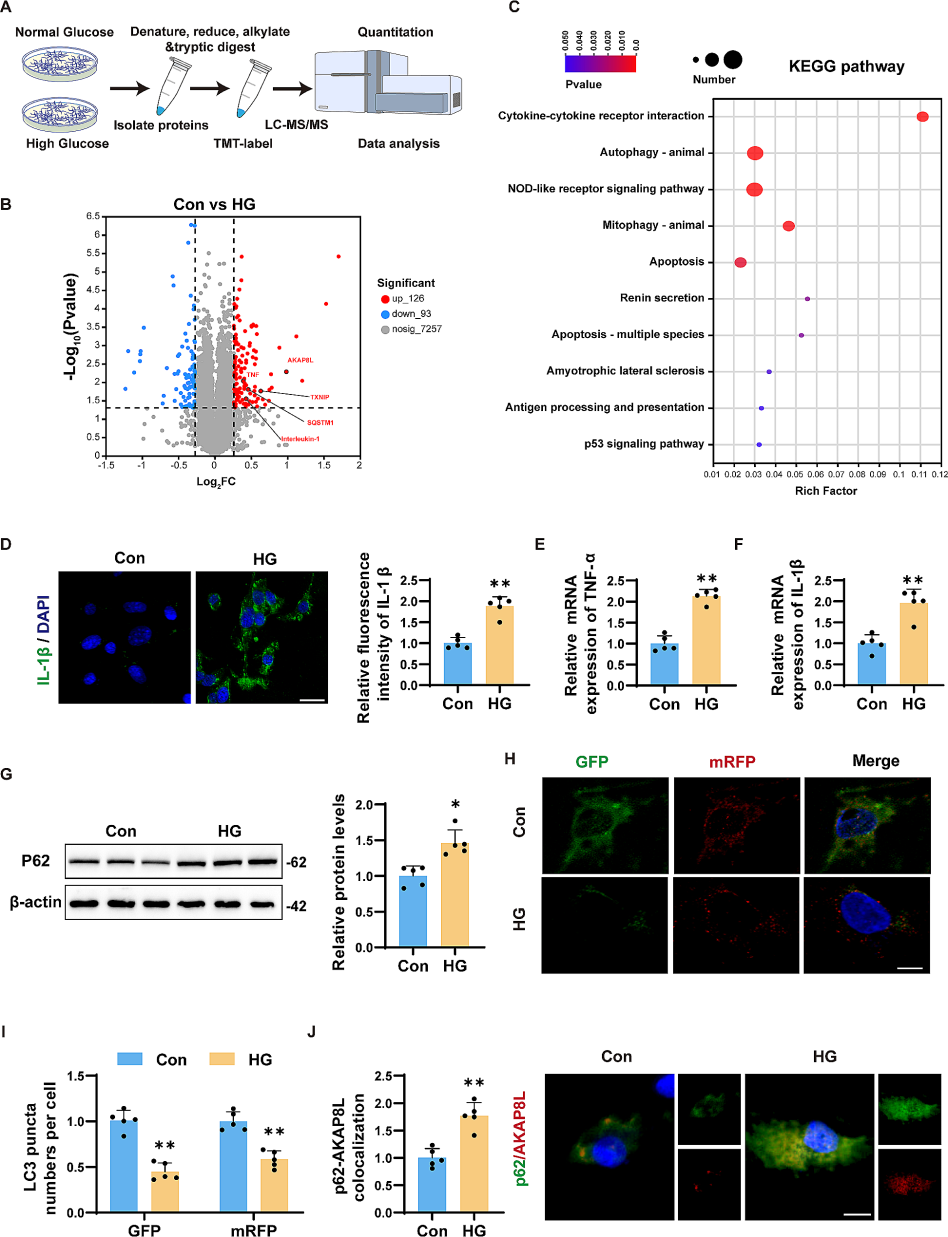
Proteomic analysis identified AKAP8L as a novel biomarker in high glucose-induced microglia. (**a**) Schematic overview of the proteomic analysis workflow in high glucose-induced microglia. (**b**) Volcano plot depicting differentially expressed proteins. (**c**) KEGG pathway enrichment analysis of differentially expressed proteins. (**d**) Representative immunofluorescence images of IL-1β with statistical quantification. Scale bar: 20 am. Relative mRNA expression of TNF-α (**e**) and IL-1β (**f**). (**g**) Representative Western blots and statistical analysis of P62 protein levels. Representative confocal microscopy images (**h**) and statistical graphs (**i**) of mRFP-GFP-LC3 puncta. Scale bar: 5 μm. (**j**) Representative immunofluorescence colocalization images and statistical graphs of p62 and AKAP8L. Scale bar: 10 μm. Data are Mean ± SD (*n* = 3–5). P-values are from unpaired Student’s t-tests. *p < 0.05 and **p < 0.01 compared to the Control group

To explore changes in protein expression and underlying mechanisms in DACI, we conducted a proteomics analysis of microglia exposed to HG. This analysis identified 126 proteins that were upregulated and 93 that were downregulated in microglia following HG treatment. The raw data for these differentially expressed proteins are presented in Supplementary Table 1. Notably, AKAP8L was among the most significantly upregulated proteins, along with an increase in proteins related to autophagy and inflammation (Fig. 1A and B). Additionally, KEGG pathway enrichment analysis highlighted the involvement of these proteins in pathways such as cytokine-cytokine receptor interactions, autophagy, NOD-like signaling cascades, mitophagy, and apoptosis (Fig. 1C). Further validation of the autophagy and inflammation pathways was performed using immunofluorescence staining, western blot, and rt-qPCR. As expected, the mRNA levels of IL-1β and TNF-α, as well as the relative fluorescence intensity specifically for IL-1β, were markedly increased in microglia exposed to HG conditions (Fig. 1D and E, and 1F). The protein level of p62 was also assessed by western blot, revealing a marked upregulation in HG-induced microglia (Fig. 1G). Autophagic flux was monitored using LC3 tandem fluorescence labeling (mRFP-GFP-LC3), which showed a decrease in LC3 puncta numbers per cells in HG-treated microglia (Fig. 1H and I). These results indicate that HG exposure disrupts autophagic flux in microglia. Intriguingly, our immunolocalization analysis showed that HG treatment enhanced the co-localization of p62 and AKAP8L in microglia, implying that AKAP8L may play a role in regulating autophagy (Fig. 1J and K).

### AKAP8L accumulation presented only in microglia, but not in astrocytes and neurons in HG-treated neural cells or in the hippocampus of STZ mice

**Fig. 2:**
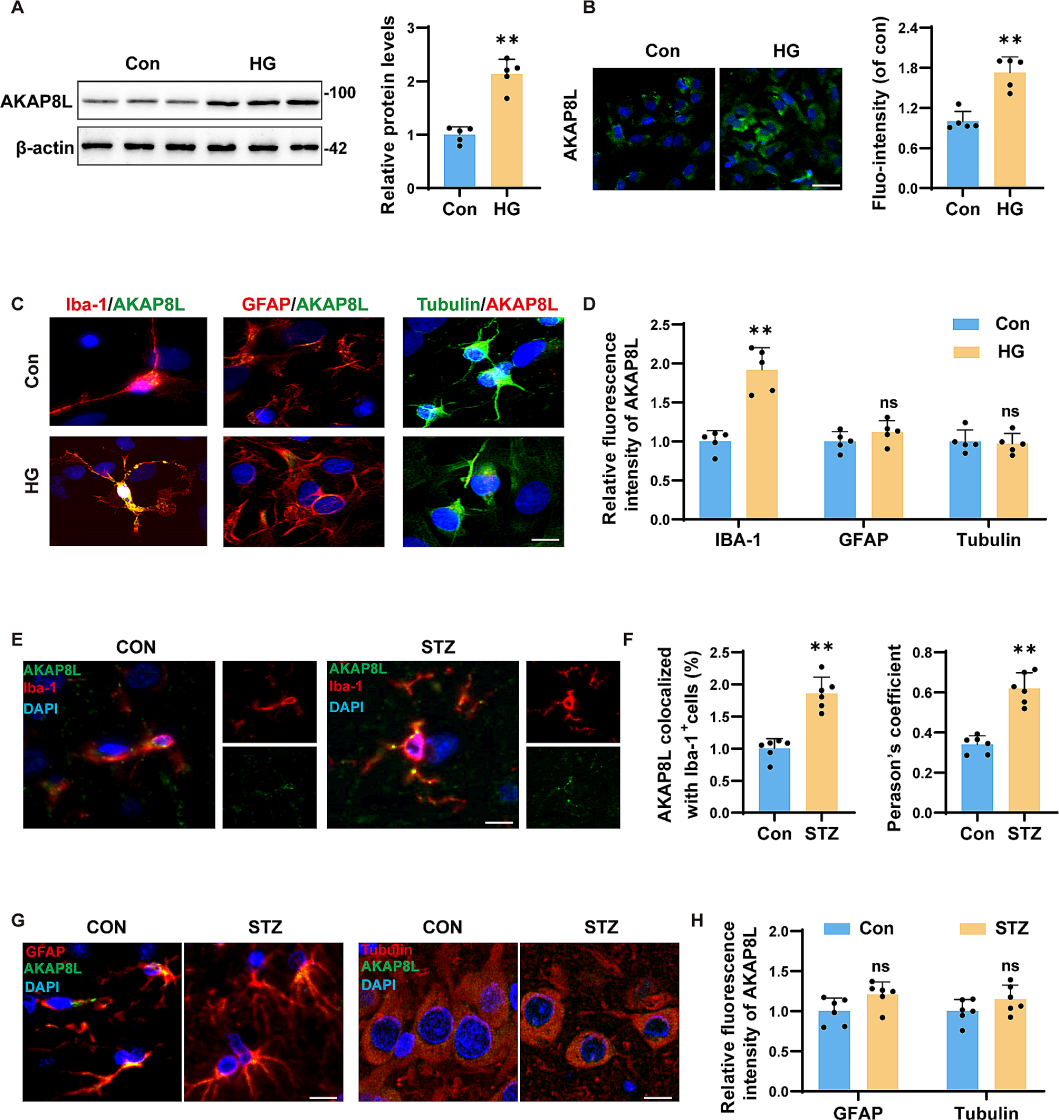
AKAP8L accumulates in microglia, absent in astrocytes and neurons in HG-treated cells and STZ mice hippocampus. (**a**) Representative Western blots and statistical analysis of AKAP8L protein levels. (**b**) Representative immunofluorescence images of AKAP8L with quantification. Scale bar: 20 μm. Colocalization of AKAP8L with microglial marker Iba-1, astrocytic marker GFAP, and neuronal marker βIII tubulin in primary neural co-cultures (**c**), with corresponding statistical analysis (**d**). Scale bar: 10 μm. (**e**-**f**) Colocalization of Iba-1 with AKAP8L in the hippocampus of mice, with quantification. Scale bar: 10 μm. (**g**-**h**) Colocalization of GFAP and βIII tubulin with AKAP8L in the hippocampus of mice, with quantification. Scale bar: 10 μm. Data are Mean ± SD (*n* = 5–6). P-values are from unpaired Student’s t-tests. *p < 0.05 and **p < 0.01 compared to the Control group

The upregulation of AKAP8L identified in the proteomic analysis was further validated using Western blot and immunofluorescence (Fig. 2A and B). To elucidate the expression profile of AKAP8L in response to co-culture and HG stimulation, we performed co-staining with specific markers: Iba-1 for microglia, GFAP for mature astrocytes, and Tubulin for neurons. Notably, AKAP8L was found to accumulate specifically in HG-treated microglia and was absent in co-cultured astrocytes and neurons (Fig. 2C and D). Consistent with these findings, similar results were observed in the hippocampal tissue of STZ-induced diabetic mice (Fig. 2E-H). Furthermore, STZ-induced diabetic mice exhibited cognitive impairment, as well as autophagy disorders and inflammation within the hippocampal region, as detailed in Supplementary Figs. 3 and 4.

### HG enhanced the interaction between AKAP8L and mTORC1 in primary microglia and BV2 cells

**Fig. 3:**
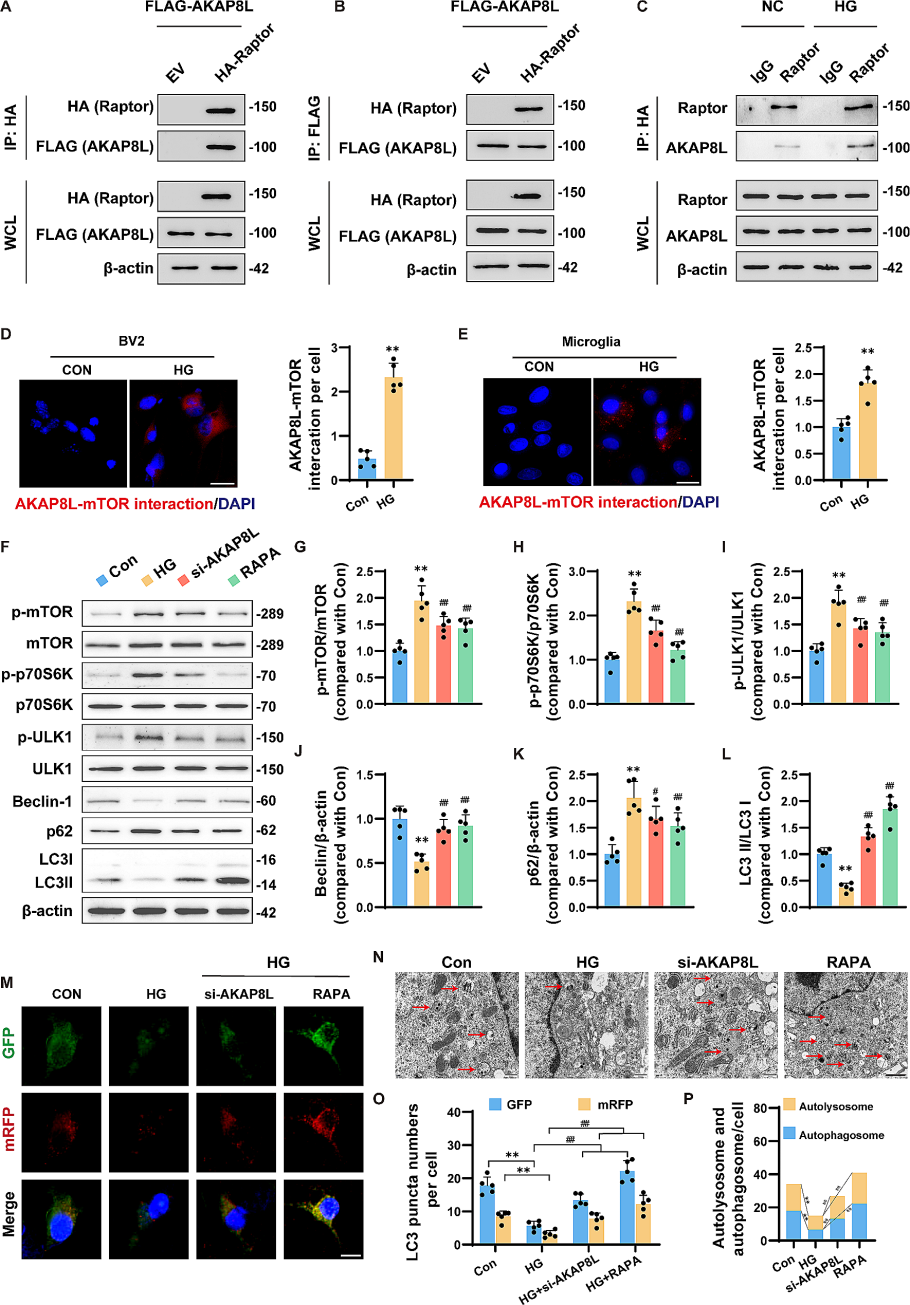
HG enhanced AKAP8L and mTORC1 interaction, and both si-AKAP8L and rapamycin mitigate HG-induced autophagy disorders in microglia. Co-immunoprecipitation of FLAG-tagged AKAP8L and HA-tagged Raptor (**a**), and vice versa (**b**), as well as the interaction between endogenous AKAP8L and Raptor (**c**). Proximity ligation assay reveals increased AKAP8L-Raptor interactions in BV2 cells (**d**) and primary microglia (**e**). Scale bar: 20 μm. (**f**) Representative blots of autophagy-related proteins. Statistical graphs of protein expression for p-mTOR/mTOR (**g**), p-p70S6K/p70S6K (**h**), p-ULK1/ULK1 (**i**), Beclin-1 (**j**), p62 (**k**), and LC3 II/LC3 I (**l**). (**m**) Immunofluorescence staining of microglial cells transfected with mRFP-GFP-LC3 adenovirus, showing punctate LC3 distribution. Scale bar: 10 μm. (**n**) Electron microscopy images of microglia reveal HG-induced autophagy alterations. Scale bar: 500 nm. (**o**) LC3 puncta numbers per cell. (**p**) Quantification of autophagosome (GFP + RFP + yellow puncta) and autolysosome (red puncta) numbers in microglia. Data are Mean ± SD (*n* = 5). P-values are determined using unpaired Student’s t-tests for two-group comparisons and one-way ANOVA with Tukey’s post hoc test for multiple comparisons. **p < 0.01 compared to the Control group. ^#^*p* < 0.05 and ^##^*p* < 0.01 compared to the STZ group

Recent literature has indicated that AKAP8L interacts with mTORC1 to regulate anabolic metabolism in renal epithelial cells [27]. However, it is not yet clear whether the same interaction occurs in microglia and whether HG enhances the interaction. To assess this interaction, we overexpressed HA-tagged Raptor and FLAG-tagged AKAP8L in BV2 cells. Co-immunoprecipitation of HA-tagged Raptor confirmed its interaction with FLAG-tagged AKAP8L under normal glucose conditions (Fig. 3A). Conversely, a reverse immunoprecipitation of FLAG-tagged AKAP8L also co-immunoprecipitated HA-tagged Raptor (Fig. 3B). Furthermore, under HG stimulation, we observed an increase in the interaction between endogenous AKAP8L and Raptor (Fig. 3C). Proximity Ligation Assay results further substantiated that HG stimulation enhanced the interaction between AKAP8L and mTORC1 in both primary microglia and BV2 cells (Fig. 3D and E).

### Si-AKAP8L and rapamycin mitigated HG-induced autophagy disorders in microglia

Si-AKAP8L markedly decreased the protein expression levels of AKAP8L, and our observations confirmed that rapamycin did not influence AKAP8L protein expression, as illustrated in Supplementary Fig. 5. Subsequently, we explored the effects of AKAP8L knockdown on mTORC1-mediated autophagy pathways. Compared to the control group, HG treatment significantly elevated the expression ratios of p-mTOR/mTOR, p-p70S6K/p70S6K, and p-ULK1/ULK1 in microglia (Fig. 3F-I). Concurrently, there was a notable decrease in Beclin-1 levels and the LC3-II/LC3-I ratio, alongside an increase in p62 levels (Fig. 3J-L), suggesting impaired autophagy under HG conditions. Moreover, the expression patterns of these proteins were reversed with si-AKAP8L and rapamycin treatment in HG-induced microglia. Consistent with these findings, mRFP-GFP-LC3 immunostaining revealed that si-AKAP8L and rapamycin significantly increased LC3 puncta formation per cell, and transmission electron microscopy also detected a restoration of autophagosome and autolysosome numbers compared to the HG group (Fig. 3M-P). Collectively, these results imply that AKAP8L knockdown and rapamycin can ameliorate autophagy impairment by inhibiting mTORC1 in HG-induced microglia.

### Si-AKAP8L and rapamycin reduced the activation of the NLRP3 inflammasome and pyroptosis in microglia induced by HG

**Fig. 4:**
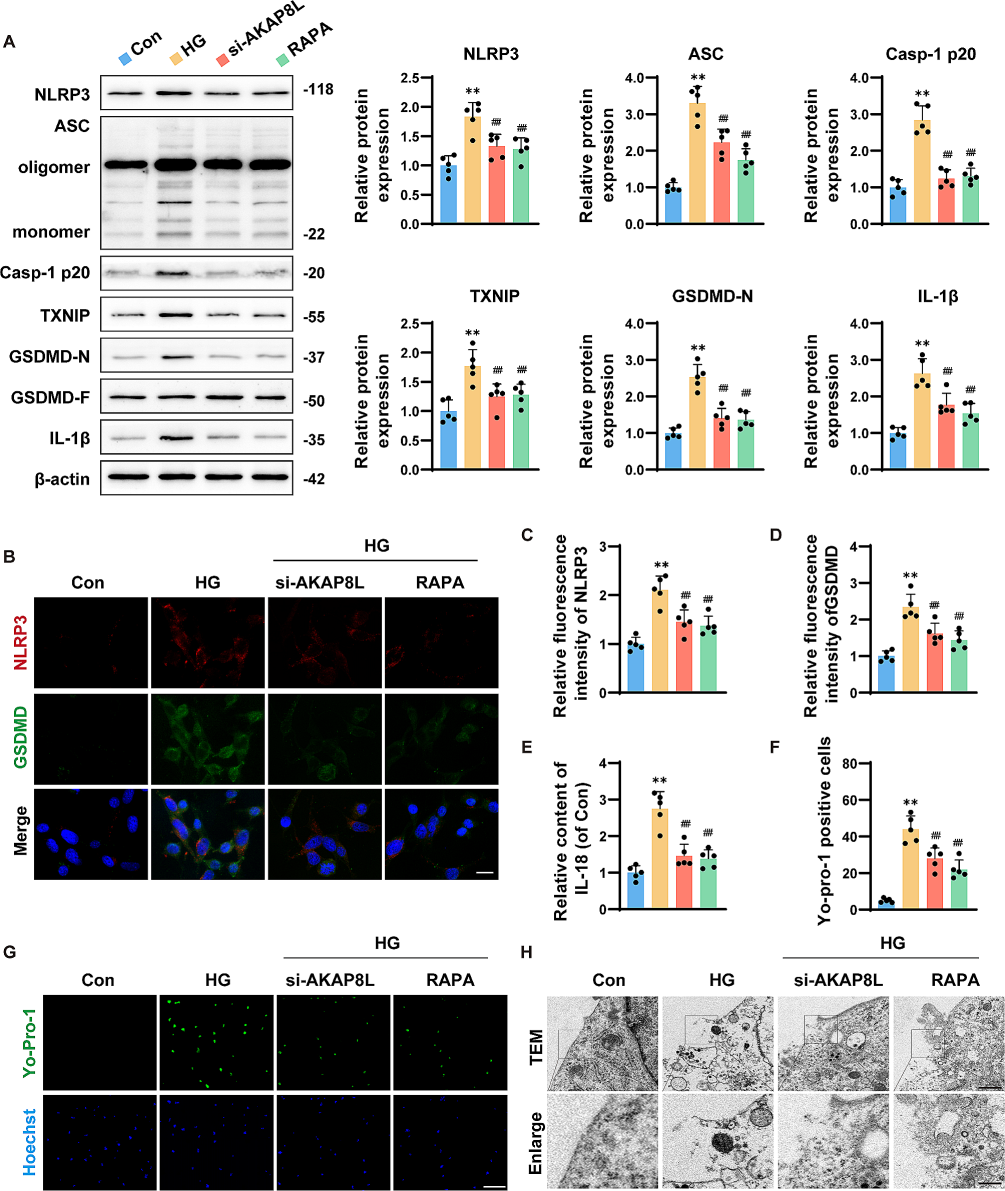
Si-AKAP8L and rapamycin alleviated high glucose-induced activation of NLRP3 inflammasomes and pyroptosis in microglia. (**a**) Representative blots and statistical analysis of NLRP3, ASC, Caspase-1 p20, TXNIP, GSDMD-N, and IL-1β in response to HG treatment. Representative immunofluorescence images (**b**) and quantification of NLRP3 (**c**) and GSDMD (**d**). Scale bar: 20 μm. (**e**) ELISA quantification of IL-18 levels. Representative images (**g**) and statistical graphs (**f**) of microglia stained with YO-PRO-1 for the analysis of pyroptotic cells. Scale bar: 50 μm. (**h**) Transmission electron microscopy images of pyroptotic cells, with a primary image at 2 μm and an inset at 1 μm scale. Data are Mean ± SD (*n* = 5). P-values are from one-way ANOVA with Tukey post hoc multiple comparisons. **p < 0.01 compared to the Control group. ^##^*p* < 0.01 compared to the STZ group

Impaired autophagy is known to exacerbate inflammation. As anticipated, HG-induced microglia showed significant upregulation of NLRP3, ASC, Caspase-1, TXNIP, GSDMD-N, and IL-1β protein levels compared to the control group, with si-AKAP8L and rapamycin treatment leading to a reduction in these protein expressions (Fig. 4A). Immunofluorescence and ELISA assays further confirmed that HG-induced microglia had elevated levels of NLRP3, GSDMD, and IL-18, which were significantly reversed by si-AKAP8L and rapamycin treatment (Fig. 4B-E). Additionally, HG-induced microglia exhibited signs of fragmented cell membranes and increased pyroptosis, as detected by Yo-Pro-1 staining and transmission electron microscopy; these abnormalities were notably rescued by si-AKAP8L and rapamycin treatment (Fig. 4G, H). These findings collectively suggest that knockdown of AKAP8L and treatment with rapamycin mitigate NLRP3 inflammasome activation and pyroptosis in HG-induced microglia via the mTORC1 pathway.

### Sh-AKAP8L and rapamycin alleviated autophagy disorders in the hippocampus of STZ mice by inhibiting mTORC1

**Fig. 5:**
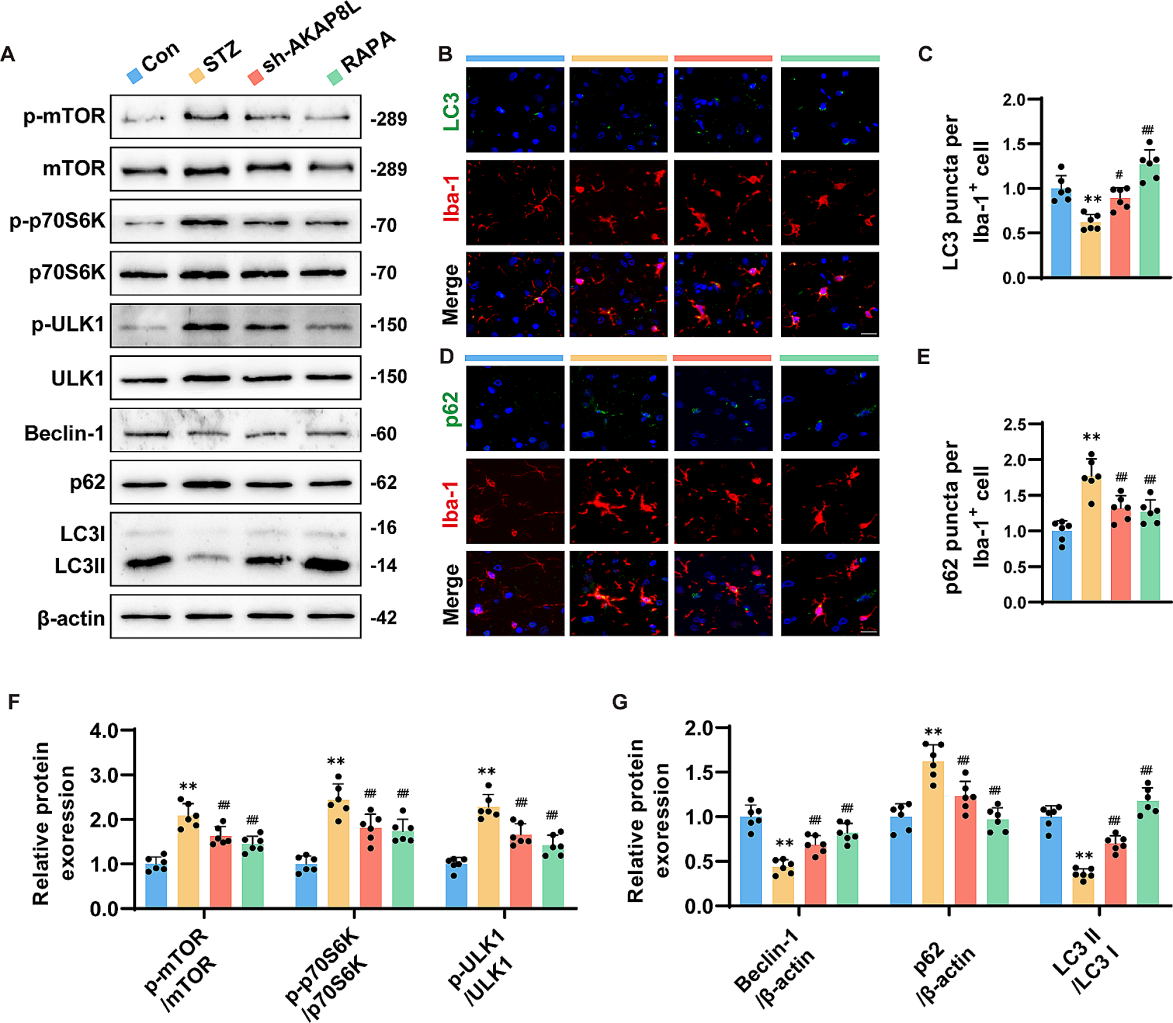
Sh-AKAP8L and rapamycin alleviated autophagy disorders in the hippocampus of STZ mice by inhibiting mTORC1. (**a**) Representative blots of p-mTOR, mTOR, p-p70S6K, p70S6K, p-ULK1, ULK1, Beclin-1, p62, LC3-II, LC3-I, and β-actin. Representative immunofluorescence images (**b**) and statistical graphs (**c**) of Iba-1 and LC3 colocalization. Scale bar: 20 μm. Representative immunofluorescence images (**d**) and statistical graphs (**e**) of Iba-1 and p62 colocalization. Scale bar: 20 μm. (**f**) Statistical graphs of protein expression for phosphorylation levels of mTOR, p70S6K, and ULK1. (g) Statistical graphs of protein expression for LC3-II/LC3-I, p62, and Beclin-1. Data are Mean ± SD (*n* = 6). P-values are from one-way ANOVA with Tukey post hoc multiple comparisons. **p < 0.01 compared to the Control group. ^#^*p* < 0.05 and ^##^*p* < 0.01 compared to the STZ group

To assess the status of autophagy within the hippocampus of STZ mice, we conducted immunofluorescence co-staining with the microglial marker Iba-1 and autophagy-associated proteins LC3 and p62. The obtained images revealed a decrease in the number of LC3 puncta per Iba-1-positive cell and an increase in the number of p62 puncta per Iba-1-positive cell in the hippocampus of STZ-treated mice when compared to controls (Fig. 5B-E). Western blotting further confirmed these findings, revealing elevated phosphorylation levels of mTOR, p70S6K, and ULK1 in the hippocampus of STZ-treated mice (Fig. 5F). Additionally, there was a notable decrease in the ratio of LC3-II to LC3-I and Beclin-1, along with an increase in p62 levels (Fig. 5G). Collectively, these results indicate that mTORC1 activation in microglia impairs autophagy in STZ-induced diabetic mice. Notably, AKAP8L knockdown and rapamycin treatment effectively reversed these autophagic deficits.

### Sh-AKAP8L and rapamycin reduced the activation of the NLRP3 inflammasome and pyroptosis in the hippocampus of STZ mice

**Fig. 6:**
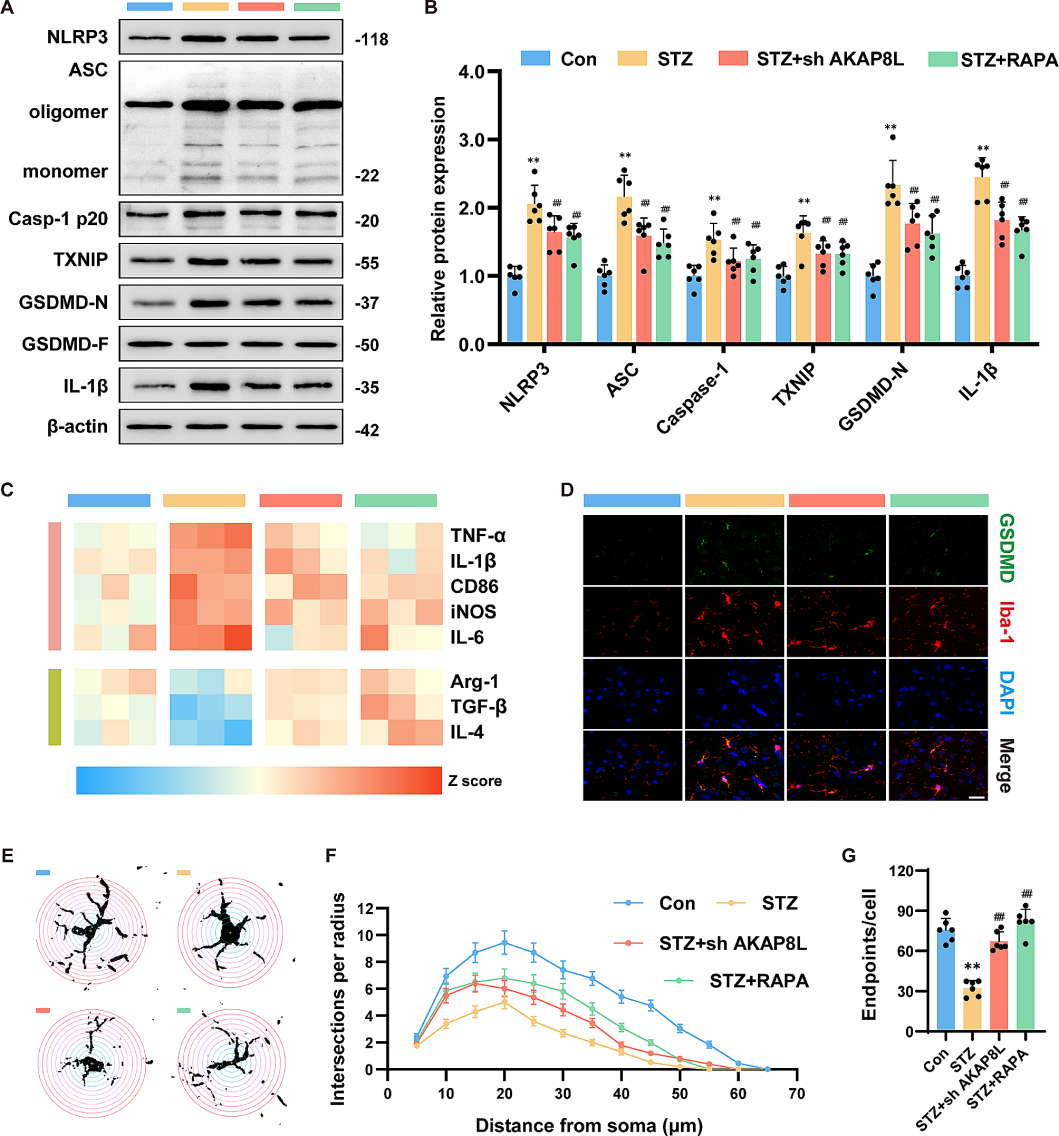
sh-AKAP8L and rapamycin mitigated NLRP3 inflammasome and pyroptosis in STZ mice via inhibiting mTORC1 activation. Representative blots (**a**) and statistical graphs (**b**) of NLRP3, ASC, Caspase-1, TXNIP, GSDMD-N, and IL-1β. (**c**) The gene expression of TNF-α, IL-1β, IL-6, CD86, iNOS, IL-4, Arg-1, and TGF-β using Z-score transformation. (**d**) Representative immunofluorescence images of GSDMD, Iba-1 and DAPI. Scale bar: 20 μm. (**e**) Representative example binary images of microglia in the hippocampus of mice. (**f**) Statistical graphs of Sholl analysis results showing the number of intersections per radius around the microglial soma. (**g**) Quantification of microglial process endpoints per cell. Data are Mean ± SD (*n* = 6). P-values are from one-way ANOVA with Tukey post hoc multiple comparisons. **p < 0.01 compared to the Control group. ^##^*p* < 0.01 compared to the STZ group

The hippocampus of STZ mice exhibited a significant upregulation of NLRP3, ASC, Caspase-1, TXNIP, GSDMD-N, and IL-1β (Fig. 6A, B). However, treatment with sh-AKAP8L and rapamycin diminished the expression of these proteins in the hippocampal tissue of STZ mice. Employing Z-score transformation, we confirmed that the expression of pro-inflammatory markers, including TNF-α, IL-1β, IL-6, CD86, and iNOS, was elevated in the hippocampus of STZ mice compared to controls (Fig. 6C). Concurrently, the expression of anti-inflammatory markers such as IL-4, Arg-1, and TGF-β was decreased in the hippocampus of STZ mice. These results highlight a significant inflammatory response in the hippocampus of STZ mice, which was markedly reduced with sh-AKAP8L and rapamycin treatment. GSDMD and the microglial marker Iba-1 colocalization was observed using immunofluorescence to assess pyroptotic status in microglia of STZ-induced mice. The visual data showed an increase in microglial pyroptosis in STZ-induced mice, which was partially attenuated by sh-AKAP8L and rapamycin treatment (Fig. 6D). Additionally, the ramified morphology of microglia, indicative of microglial inflammation, was assessed using Sholl analysis (Fig. 6E-G). Microglia in the control group exhibited a highly branched, resting state morphology, whereas those in STZ mice adopted an amoeboid shape and showed reduced endpoints per cell. Notably, sh-AKAP8L and rapamycin treatment restored the ramified morphology and increased endpoints per cell in microglia of STZ mice. In summary, these findings suggest that sh-AKAP8L and rapamycin can alleviate neuroinflammation and pyroptosis in the microglia of STZ mice.

### Sh-AKAP8L and rapamycin protected against neuronal damage and cognitive impairment in STZ mice

**Fig. 7:**
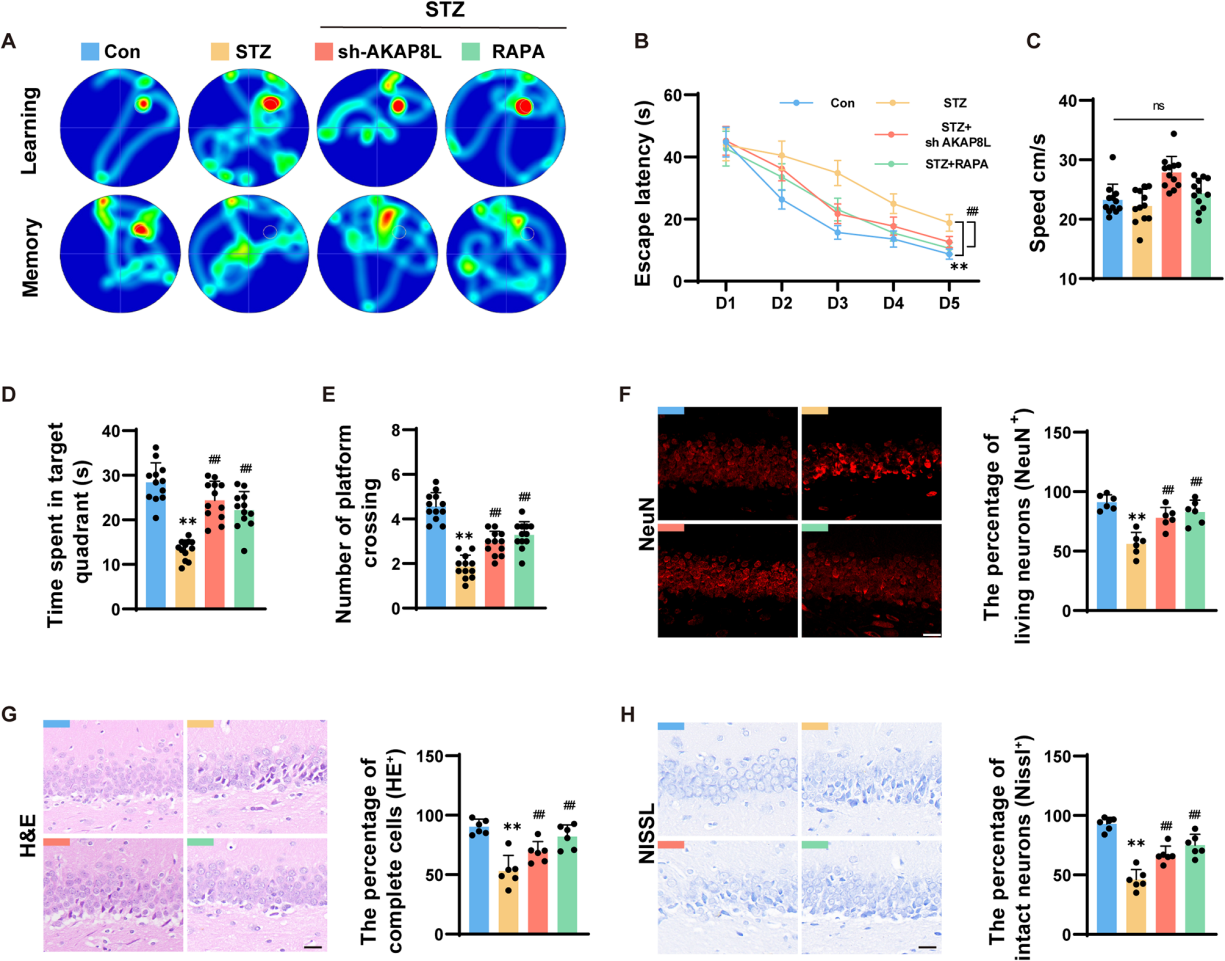
Sh-AKAP8L and rapamycin protected against neuronal damage and cognitive impairment in STZ mice. (**a**) Swimming trajectory images of the CON, STZ, STZ + sh-AKAP8L, and STZ + RAPA groups in the MWM test. (**b**) Escape latency during the training session. (**c**) Average speed of each group. (**d**) Statistical graphs of the time spent in the target quadrant. (**e**) Number of crossings over the original platform location. (**f**) Images and statistical graphs of NeuN immunofluorescence staining in the hippocampal CA1 region, representing the rate of viable neurons. (**g**) Images and statistical graphs of H&E staining in the hippocampal CA1 region, representing the rate of intact cells. (**h**) Images and statistical graphs of Nissl staining in the hippocampal CA1 region, representing the rate of intact neurons. Scale bar: 20 μm. Data are Mean ± SD (*n* = 6 or 12). P-values are from one-way or two-way ANOVA with Tukey post hoc multiple comparisons. **p < 0.01 compared to the Control group. ^##^*p* < 0.01 compared to the STZ group

To evaluate the impact of AKAP8L knockdown on cognitive function in STZ-induced diabetic mice, we employed the Morris water maze test for assessing spatial learning and memory (Fig. 7A-E). Compared to control mice, STZ mice exhibited significantly prolonged escape latencies, reduced time in the target quadrant, and fewer platform crossings. However, mice in the sh-AKAP8L + STZ and RAPA + STZ groups displayed shorter escape latencies, increased time in the target quadrant, and more platform crossings compared to those in the STZ group alone. These findings suggest that while cognitive and memory functions are impaired in STZ mice, they can be significantly improved with sh-AKAP8L knockdown and rapamycin treatment.

Furthermore, to assess neuronal integrity in the hippocampus—a region pivotal for cognition and memory—we performed NeuN, HE, and Nissl staining on hippocampal tissue samples from STZ mice (Fig. 7F-H). Microscopic analysis revealed a marked reduction in the percentage of intact cells and viable neurons in the hippocampal CA1 region of the STZ group when compared to the control group. This neuronal degeneration was ameliorated by sh-AKAP8L knockdown and rapamycin treatment. Comparable findings were observed in both the CA3 region and the DG region of the hippocampus (Supplementary Fig. 6). Collectively, these results indicate that STZ induces hippocampal and neuronal damage, which can be attenuated by sh-AKAP8L knockdown and rapamycin treatment.

## Discussion

**Fig. 8:**
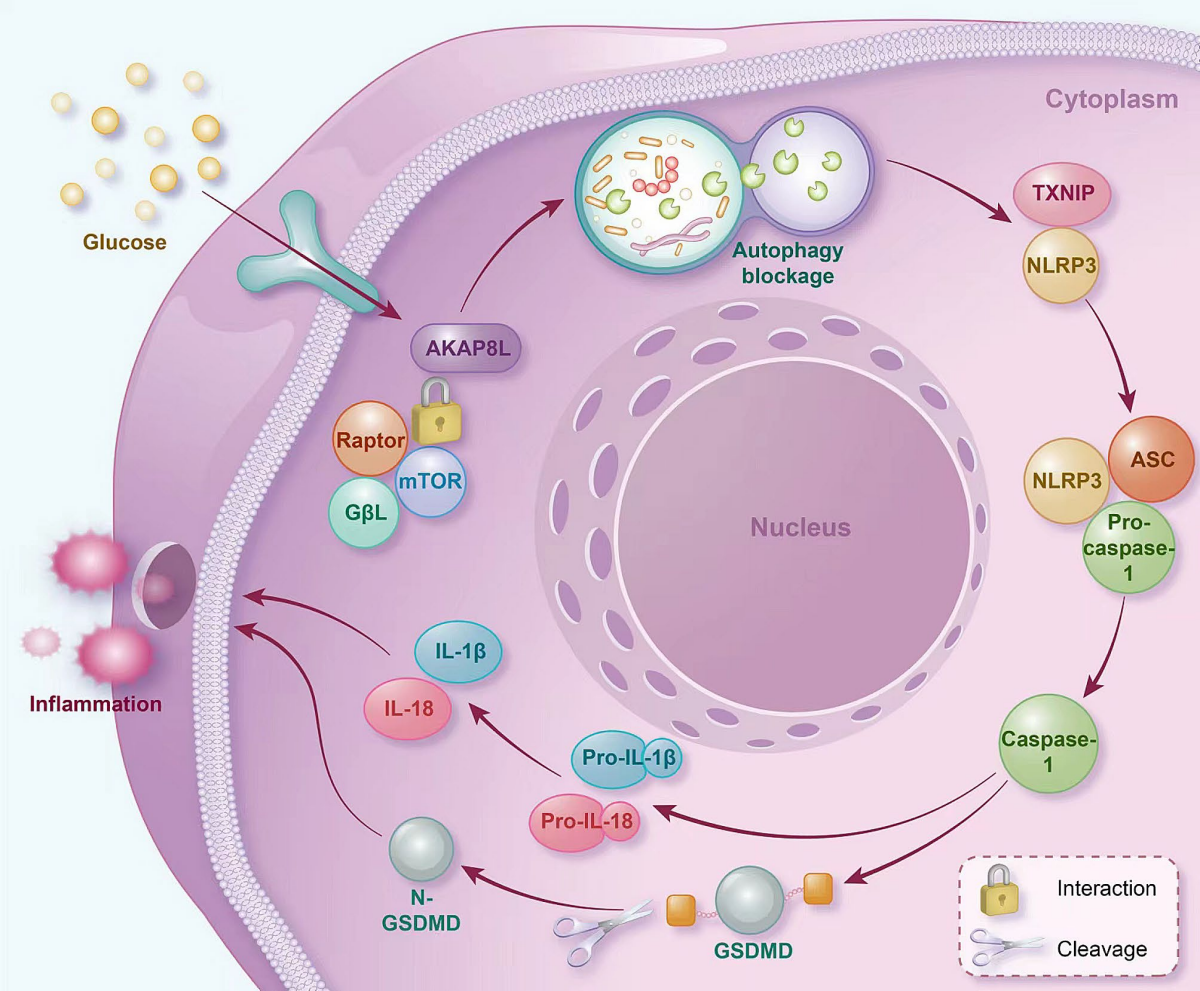
The mechanism diagram of AKAP8L in the potential pathogenesis of DACI

In the present study, proteomic analysis revealed a marked upregulation of AKAP8L in HG-treated microglia, accompanied by aberrant expression of autophagy and inflammation markers. Notably, the accumulation of AKAP8L was specific to HG-treated microglia, with no observed changes in co-cultured astrocytes or neurons, a pattern that was mirrored in STZ-induced diabetic mice. Further studies through co-immunoprecipitation and PLA indicated that the elevated AKAP8L in HG-treated microglial cells interacts with the mTORC1. In the STZ mouse model, we demonstrated that both AKAP8L knockdown and rapamycin treatment significantly enhanced cognitive function, as evidenced by improved performance in the Morris water maze, and reduced microglial activation. Moreover, these interventions effectively suppressed mTORC1 signaling, normalized autophagic flux, mitigated neuroinflammation, and decreased pyroptosis. The involvement of AKAP8L in the potential pathogenesis of DACI is illustrated in Fig. 8.

Proteomics stands as a cornerstone in diabetes research, offering unparalleled insights into the molecular mechanisms underlying the disease. However, there have been no reports on proteomic studies of microglial cells treated with HG to date. The current proteomic analysis, through KEGG pathway enrichment analysis, has revealed disrupted pathways that include the cytokine-cytokine receptor interaction, autophagy, NOD-like receptor signaling cascade, mitophagy, and apoptosis, among others. The findings, in concordance with existing literature [28, 29], underscore the pivotal roles of autophagy and inflammation in the pathogenesis of DACI. Additionally, disruptions in autophagy and inflammation have been observed in the Alzheimer’s disease (AD) brain, occurring well in advance of any observable clinical symptoms of the condition [30]. The volcano plot further revealed that the expression of proteins such as AKAP8L, TNF, TXNIP, SQSTM1, and IL1 was significantly upregulated, with AKAP8L being identified as a novel marker for the first time.

AKAP8L, a member of the A-kinase anchor protein (AKAP) family renowned for its role in localizing protein kinase A (PKA) to distinct subcellular sites [31], has emerged as a focal point in research exploring its contributions to tumor development and a spectrum of biological functions. These include the regulation of RNA splicing, histone phosphorylation, and the identification of prognostic indicators in cancer [32, 33]. In our current research, we have identified AKAP8L as a pivotal factor in the pathogenesis of DACI. Prior research has also documented significant increases in AKAP8L levels in the plasma of patients afflicted with diabetic liver disease, underscoring the substantial involvement of AKAP8L in the complications associated with diabetes [34]. Remarkably, the aggregation of AKAP8L was uniquely observed in microglia subjected to HG conditions, with no detectable alterations in co-cultured astrocytes and neurons, a phenomenon consistent with patterns witnessed in STZ-diabetic murine models. The findings indicate that microglia could play a pivotal role in initiating brain injury associated with diabetes. Activation of these cells leads to an escalation of neuronal apoptosis in the hippocampal region of STZ-diabetic mice, thereby aggravating the neurodegenerative cascade [35]. Focusing on the hippocampus, which mainly comprises the CA1, CA3, and DG — all crucial for cognitive function [36] — we reveal a significant reduction in viable neurons in the STZ-affected hippocampal regions compared to controls. These neuronal damages were found to be attenuated by sh-AKAP8L knockdown and rapamycin treatment, presenting potential therapeutic strategies for DACI.

Recent literature has revealed that AKAP8L can interact with mTORC1 to regulate anabolic metabolism in renal epithelial cells [27]. However, it is not yet clear whether the same interaction occurs in microglia. Our findings indicate that HG conditions specifically enhance the interaction between AKAP8L and mTORC1 in microglial cells, which may be crucial for the development of DACI. mTORC1 represents a pivotal target for modulating the metabolic profile and functionality of microglia, and may be a promising avenue for enhancing cognitive abilities [37, 38]. The mTORC1 inhibitor rapamycin is known to mitigate the advancement of neurodegenerative disorders by bolstering autophagy, alleviating chronic inflammation, diminishing β-amyloid (Aβ) accumulation, and curbing the hyperphosphorylation of tau protein [39]. Nevertheless, rapamycin’s adverse side effects, such as immune suppression, oral ulcers, and hyperglycemia, are linked to the inhibition of mTORC2 [40, 41]. Therefore, the selective targeting of mTORC1 through specific drugs is of considerable importance [42]. AKAP8L specifically engages with the mTORC1 subunit Raptor. Our findings demonstrate that AKAP8L inhibition replicates the effects of rapamycin, encompassing heightened autophagy, subdued neuroinflammation, and amelioration of cognitive function in STZ-diabetic rodents. Consequently, AKAP8L may emerge as a novel target for the selective inhibition of mTORC1, offering a potential therapeutic strategy for DACI.

Autophagy, a lysosomal-dependent cellular process of catabolism, is crucial for the clearance of protein aggregates and damaged organelles, thus maintaining cellular homeostasis. In microglia, impaired autophagy is recognized as a significant contributor to cognitive decline [30, 43]. Studies have shown that cognitive deficits are exacerbated in STZ-induced diabetic mice when autophagy is inhibited by 3-methyladenine (3-MA) [44]. Metformin has been shown to enhance cognitive capabilities by stimulating hippocampal autophagy [45, 46]. The mTORC1 plays a pivotal role in regulating autophagy through its influence on the kinase ULK1 [47]. In our investigation, we found that the suppression of mTOR and the subsequent promotion of autophagy by ULK1, achieved either through sh-AKAP8L or rapamycin, significantly improved cognitive function in STZ-induced diabetic mice. Deficiency in autophagy within microglia is known to exacerbate inflammation, which is another critical factor contributing to neurodegenerative processes and cognitive decline [48–50]. The NLRP3 inflammasome plays a prominent role in neuroinflammation across a spectrum of neurological disorders [51]. Furthermore, NLRP3 is directly associated with the process of pyroptosis, a form of programmed cell death. In line with previous findings [28], our research revealed that HG conditions stimulate pyroptosis by increasing NLRP3 expression. Additionally, we observed that the inhibition of mTORC1 using si-AKAP8L and rapamycin effectively reduced the activation of the NLRP3 inflammasome, the production of inflammatory mediators, and the incidence of pyroptosis.

## Conclusions

In summary, proteomic analysis has uncovered a significant upregulation of AKAP8L in microglia exposed to HG conditions, which was associated with altered expression patterns of autophagy and inflammation-related proteins. Importantly, AKAP8L accumulation was exclusive to HG-treated microglia, with no changes detected in co-cultured astrocytes or neurons. The research further revealed that microglia subjected to HG environments enhance the interaction between AKAP8L and mTORC1. Moreover, the study demonstrated that interventions targeting mTORC1 by AKAP8L knockdown and rapamycin could mitigate cognitive deficits in STZ-induced diabetic mice by normalizing autophagy and reducing neuroinflammation. These findings suggest that the modulation of AKAP8L could represent a promising therapeutic approach for addressing DACI.

### Electronic supplementary material

Below is the link to the electronic supplementary material.

Supplementary Material 1

Supplementary Material 2

Supplementary Material 3

## Data Availability

No datasets were generated or analysed during the current study.

## Abbreviations

AKAP: A-kinase anchor protein
AKAP8L: A-kinase anchor protein 8-like
ANOVA: Analyses of variance
Arg1: Arginase-1
ASC: Apoptosis-associated speck-like protein containing a CARD
ATP5PB: ATP Synthase Peripheral Stalk-Membrane Subunit B
Aβ: β-amyloid
C3: Complement C3
C3aR: Complement C3a Receptor
C6: Complement C6
CD86: Cluster of differentiation 86
CRELD1: Cysteine rich with EGF like domains 1
DACI: Diabetes-associated cognitive impairment
GDF-15: Growth differentiation factor-15
GFAP: Glial fibrillary acidic protein
GO: Gene Ontology
GSDMD: Gasdermin D
HE: Hematoxylin-eosin
HG: High glucose
HP: Haptoglobin
Iba-1: Ionized calcium-binding adapter molecule 1
IL: Interleukin
iNOS: Inducible nitric oxide synthase
KEGG: Kyoto Encyclopedia of Genes and Genomes
LC3: Light Chain 3
MIC-1: Macrophage inhibitory cytokine-1
mTORC1: Mechanistic target of rapamycin complex 1
mTORC2: Mechanistic target of rapamycin complex 2
MWM: Morris Water Maze test
NDRG2: N-Myc downstream regulated gene 2
NF-κB: Nuclear factor kappa-B
NLRP3: NOD-like receptor thermal protein domain associated protein 3
NR: Non-Redundant Protein Database
p62/SQSTM1: Sequestosome-1
p70S6K: The 70 kDa ribosomal protein S6 kinase
PKA: Protein kinase A
RAPA: Rapamycin
ROC: Receiver Operating Characteristic
SERPIND: Serpin domain-containing protein
siRNA: small interfering RNA
STZ: Streptozotocin
TGF-β: Transforming growth factor-β
TNF-α: Tumor necrosis factor-α
TXNIP: Thioredoxin interacting protein
ULK1: Unc-51 like autophagy activating kinase 1
VNN2: Vanin 2
3-MA: 3-methyladenine
