# Supplementary material for: Microglial AKAP8L: a key mediator in diabetes-associated cognitive impairment via autophagy inhibition and neuroinflammation triggering
Source: J Neuroinflammation. 2024 Jul 20;21:177. doi: 10.1186/s12974-024-03170-z (PMC11264944; doi:10.1186/s12974-024-03170-z)
Supplement: Supplementary file 2 — Supplementary Material 2 [file 12974_2024_3170_MOESM2_ESM.pdf]

Fig1G
Anti-p62

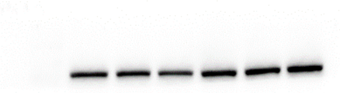

Anti-β-actin

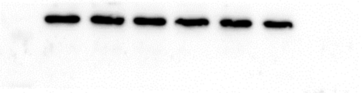

Fig2A
Anti-AKAP8L

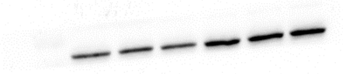

Anti-β-actin

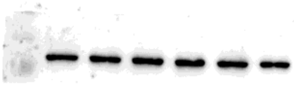

Fig3A
IP: Anti-HA

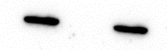

Anti-Flag

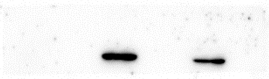

WCL: Anti-HA

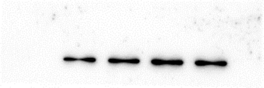

Anti-Flag

Anti-β-actin

Fig3B

IP: Anti-HA

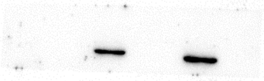

Anti-Flag

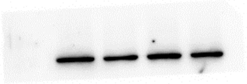

WCL: Anti-HA

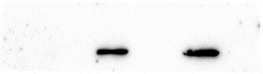

Anti-Flag

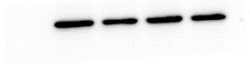

Anti-β-actin

Fig3C

IP: Anti-Raptor

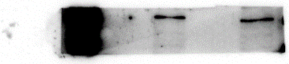

Anti-AKAP8L

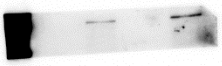

WCL: Anti- Raptor

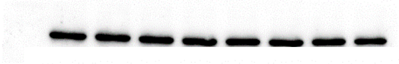

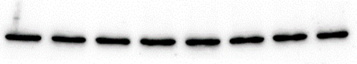

Anti-AKAP8L

Anti-β-actin

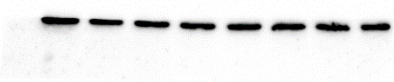

Fig3F

p-Mtor

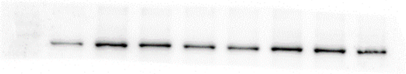

mTOR

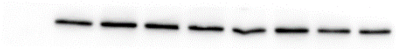

p-p70s6k

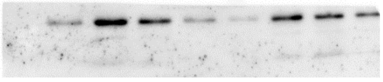

p70s6k

p-ULK1

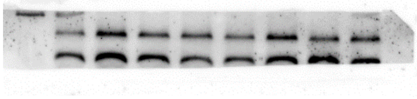

ULK1

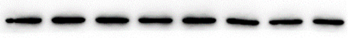

Beclin1

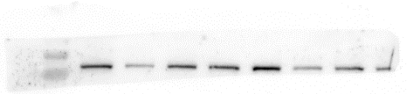

P62

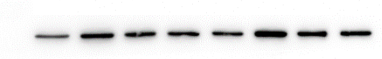

LC3

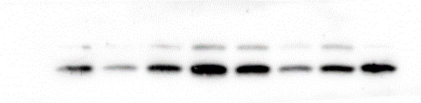

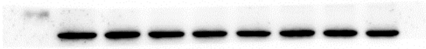

β-actin

Fig4A
NLRP3

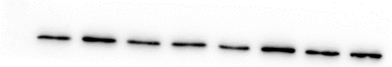

ASC

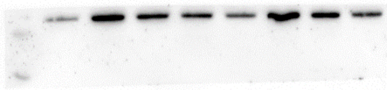

Caspase1

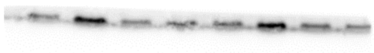

TXNIP

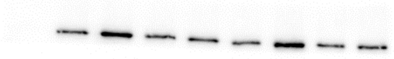

GSDMD-N

GSDMD-F

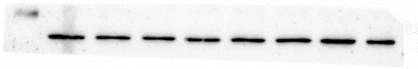

IL-1β

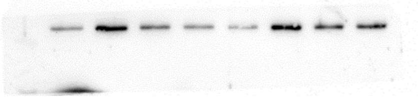

β-actin

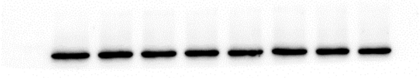

Fig5A

p-mTOR

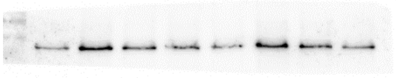

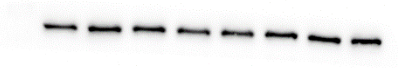

mTOR

p-p70s6k

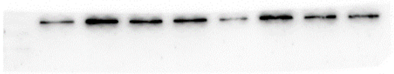

p70s6k

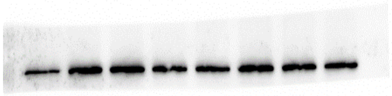

p-ULK1

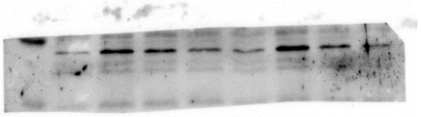

ULK1

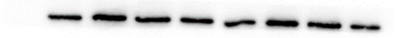

Beclin1

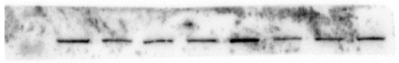

P62

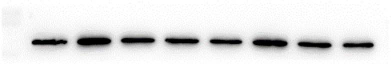

LC3

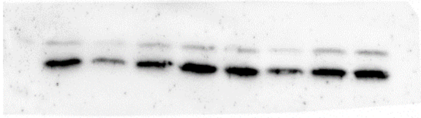

β-actin

Fig6A

NLRP3

ASC

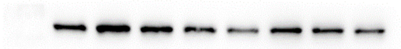

Caspase1

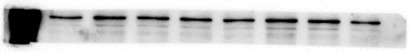

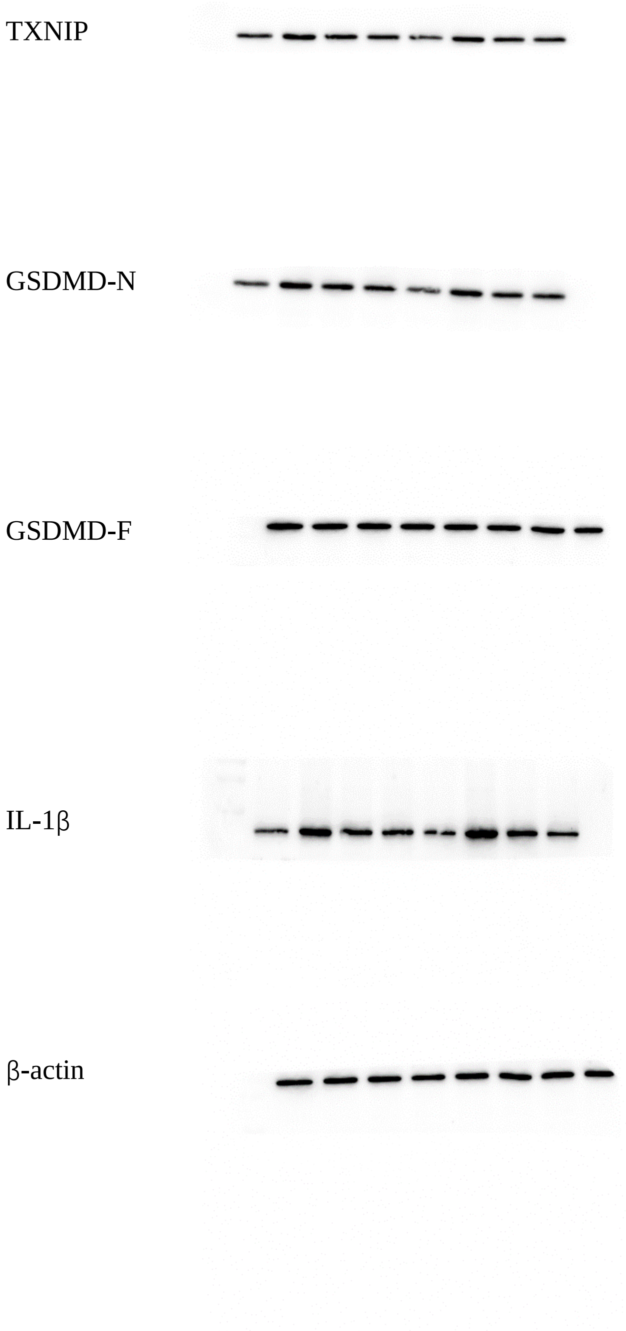
TXNIP
GSDMD-N
GSDMD-F
IL-1β
β-actin
