# Supplementary material for: Microglial AKAP8L: a key mediator in diabetes-associated cognitive impairment via autophagy inhibition and neuroinflammation triggering
Source: J Neuroinflammation. 2024 Jul 20;21:177. doi: 10.1186/s12974-024-03170-z (PMC11264944; doi:10.1186/s12974-024-03170-z)

Supplementary Figure. 1

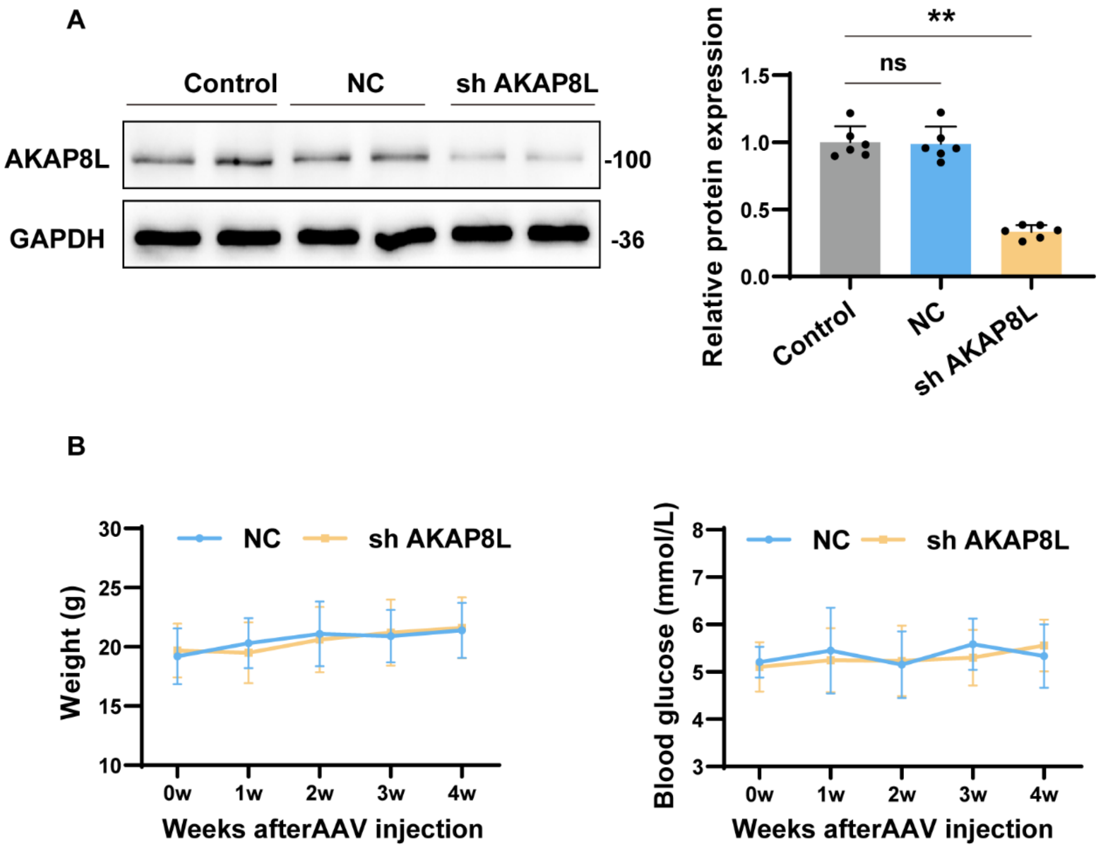

Supplementary Figure. 2

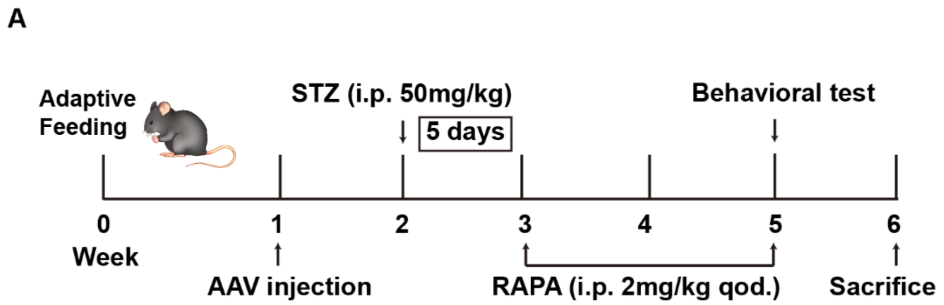

Supplementary Figure. 3

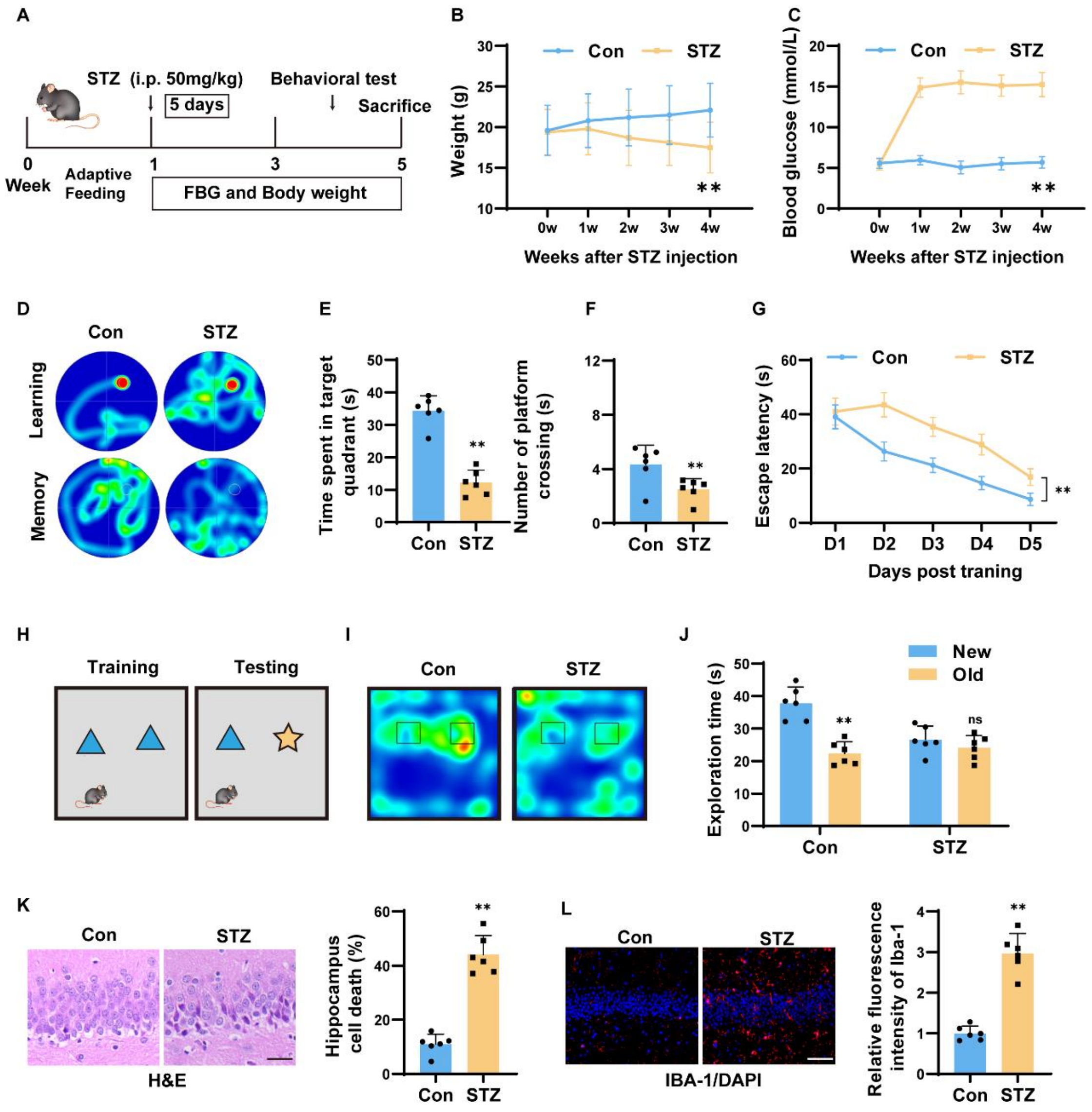

Supplementary Figure. 4

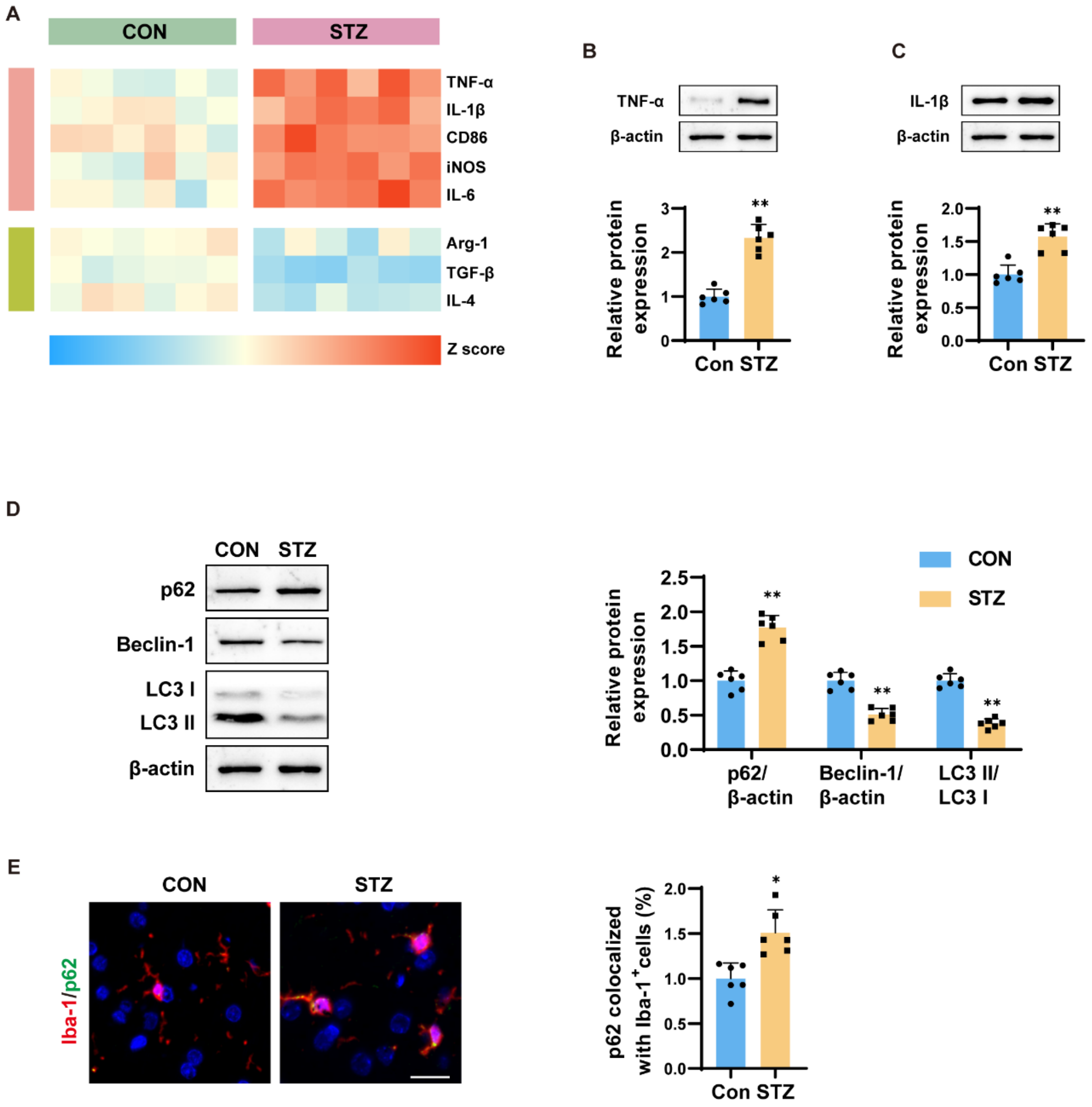

Supplementary Figure. 5

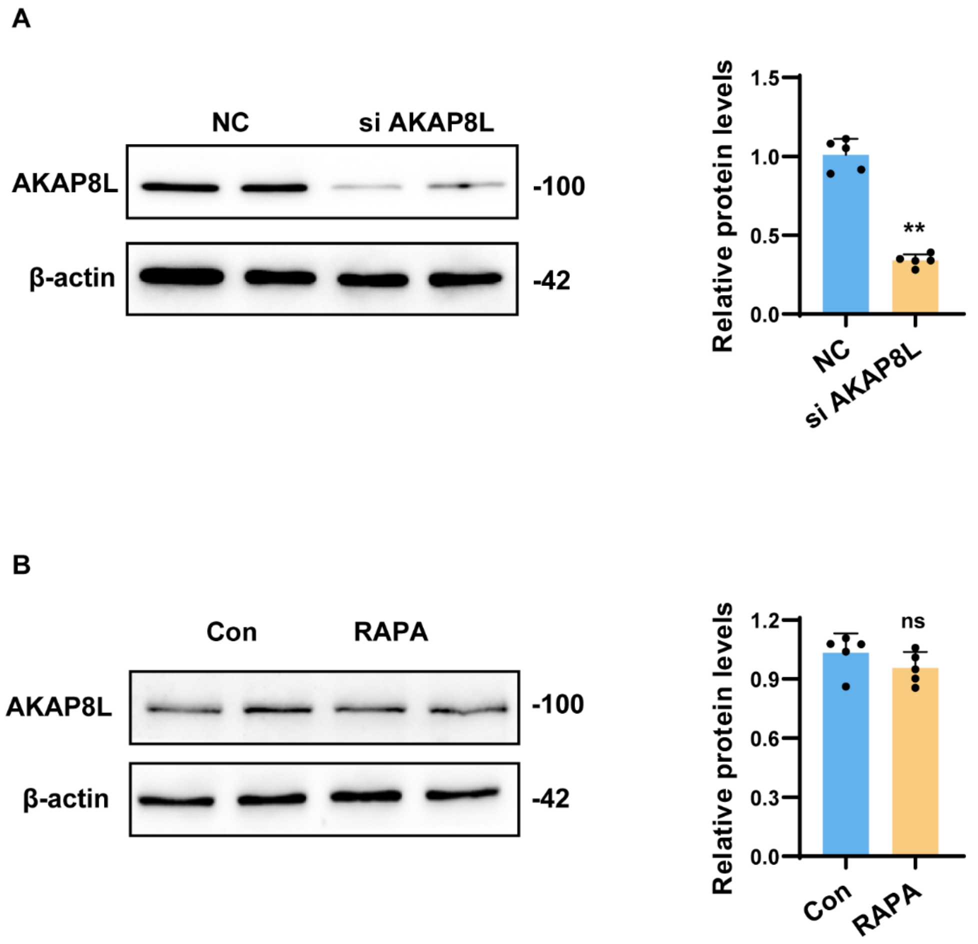

Supplementary Figure. 6

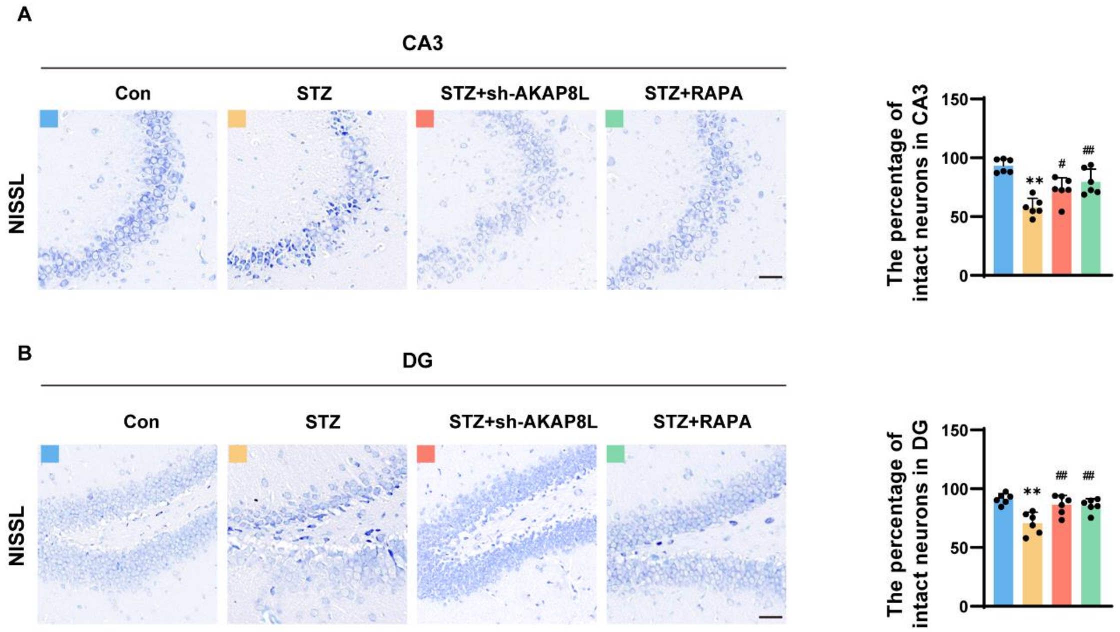

Supplement: Supplementary file 3 — Supplementary Material 3 [file 12974_2024_3170_MOESM3_ESM.pdf]
